# *Click’n lock*: rapid exchange between unsymmetric tetrazines and thiols for reversible, chemoselective functionalisation of biomolecules with on-demand bioorthogonal locking†

**DOI:** 10.1039/d3cb00062a

**Published:** 2023-07-20

**Authors:** Katerina Gavriel, Dustin C. A. van Doeselaar, Daniëlle W. T. Geers, Kevin Neumann

**Affiliations:** a Systems Chemistry Department, Institute for Molecules and Materials, Radboud University Nijmegen Heyendaalseweg 135 6525 AJ Nijmegen The Netherlands kevin.neumann@ru.nl

## Abstract

The late-stage functionalisation and diversification of complex structures including biomolecules is often achieved with the help of click chemistry. Besides employing irreversible click-like reactions, many synthetic applications benefit from reversible click reaction strategies, so called de-/trans-click approaches. Yet, the combination of both, reversible and irreversible click chemistry – while still respecting the stringent criteria of click transformations – remains so far elusive for modifications of biomolecular structures. Here, we report *click’n lock* as a concept that enables reversible click reactions and on-demand locking of chemical entities, thus switching from reversible to irreversible modifications of complex biomolecules. For this purpose, we employ the tetrazine–thiol exchange (TeTEx) reaction as a fully traceless click reaction with second order rate constants *k*_2_ higher than 2 M^−1^ s^−1^ within aqueous environments. Employing TeTEx as a reversible click reaction for the chemoselective modification of biomolecules is made possible by the use of 3,6-disubstituted 1,2,4,5-tetrazines bearing a single sulfide residue. The inherent reactivity of tetrazines towards inverse electron demand Diels–Alder (IEDDA) reactions allows to stabilize the clicked structure, switching from reversible to irreversible systems (*click’n lock*).

## Abstract 2

*Click’n lock* describes a reaction system capable of switching on-demand from reversible to irreversible click reactions and is based on the chemoselective tetrazine–thiol exchange. Here, *click’n lock* is applied for dynamic modifications of peptides.

## Graphical abstract

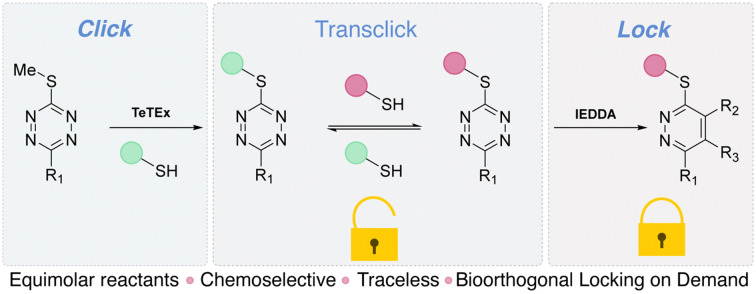

## Introduction

The principle of modifying unprotected molecular entities to enhance complexity using click chemistry is one of the most recent advancements in the field of synthetic chemistry. Although only defined in 2001, a vast range of click reactions are reported that enable precision synthesis and late-stage modification of complex structures, including folded proteins and even living cells.^1–3^ Yet, the criteria of click and click-like reactions – that are stoichiometric reagents, water-compatibility, high conversions and high degrees of chemo- and regioselectivity – make them not only attractive tools for the synthesis of small molecules and chemically tailored biomolecules but also for the fabrication of advanced materials.^3–5^ Among the most employed reactions are the copper-catalyzed (CuAAC) and strain-promoted 1,3-dipolar azide–alkyne (SPAAC) cycloadditions, Diels–Alder and inverse electron demand Diels–Alder (IEDDA) cycloadditions, conjugate additions of thiols and thiol–ene reactions.^1,6–13^

**Fig. 1:**
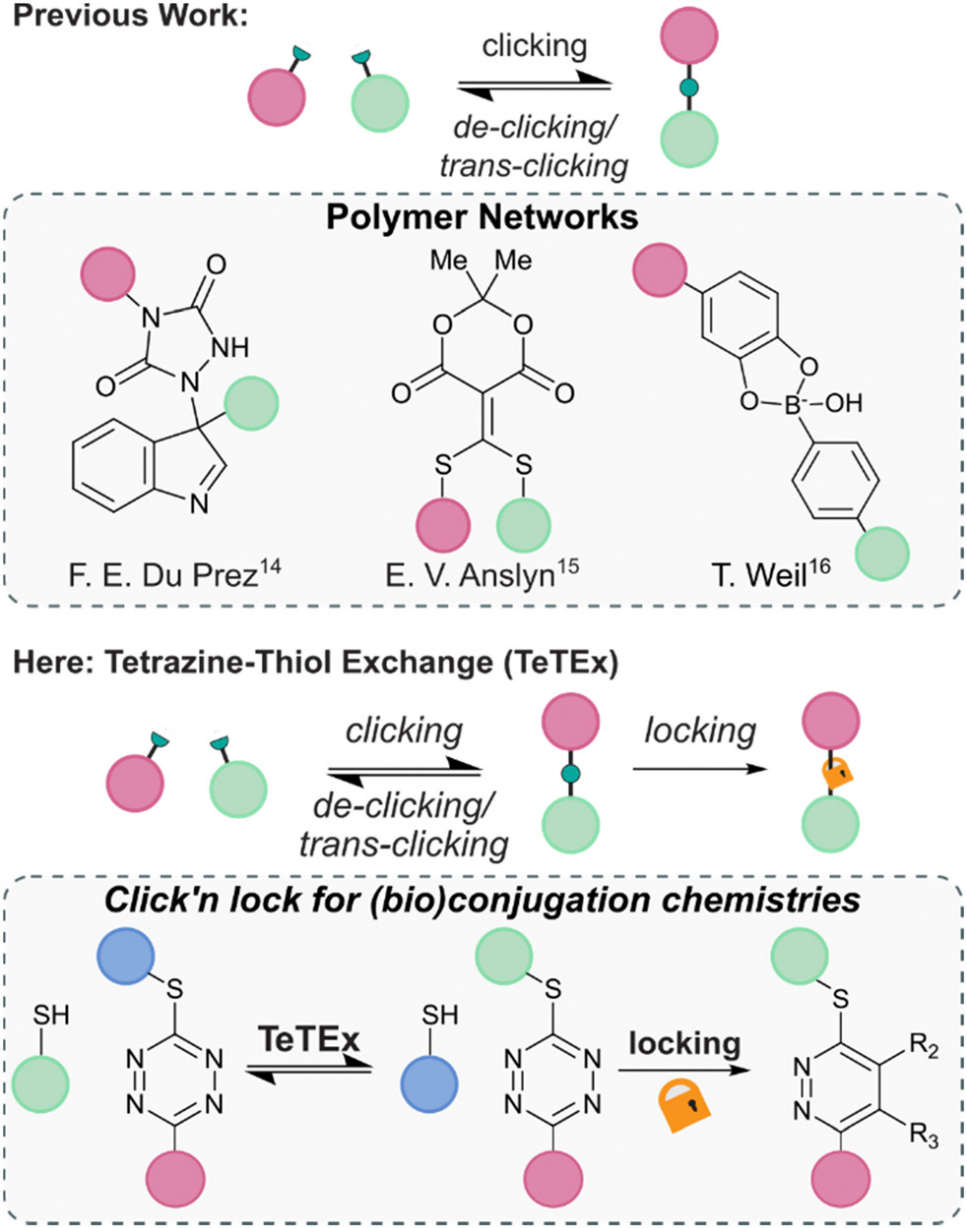
Previous reports of reversible conjugation chemistries found in the context of polymer networks. From left to right; ene adduct of 1,2,4-triazoline-3,5-diones with indoles, thiols added to a Meldrum's acid-based conjugate acceptor, and click chemistry adduct between boronic acids and diols.^14–16^ Here, we report tetrazine–thiol exchange TeTEx as a fully reversible click reaction between non-symmetric thiol functional tetrazines and thiol substrates for the modification of functionalized biomolecules. On-demand locking with a bioorthogonal stimulus provides a switch from reversibility to irreversibility.

In recent years, increasing attention was given to the development of reversible click reactions. De-click reactions result to the on-demand unlinking of the two click reaction partners, while trans-click reactions substitute one of the two reaction partners with another.^14,15^ Selectively unlinking molecules into defined fragments offers numerous applications in, amongst others, chemical biology for example as affinity purification tool and drug release. In 2014, the group of Du Prez reported in pioneering work the temperature-responsive, fully reversible ene-type click reaction of 1,2,4-triazoline-3,5-diones with indoles (Fig. 1).^14^ Following this report, the group of Anslyn demonstrated that a thiol added to a Meldrum's acid-based conjugate acceptor could be fully released upon addition of another thiol.^15^ Since then, some more examples of de-click reactions were reported including the pH-dependent dynamic covalent chemistry between boronic acids with diols and salicylhydroxamates.^16^

Combining the features of irreversible click reactions and reversible de-/trans-click reactions in a single reaction system for bioconjugation applications would provide chemists maximal control over chemical structures and complexity with temporal resolution. While this concept was reported for dynamic covalent chemistries with applications in polymer networks, the combination of click and de-/trans-click reactions remains elusive in the broader context of synthetic chemistry – in particular for bioorthogonal systems and bioconjugations.^17,18^ This is because of the inherent challenge to identify suitable reaction systems that display sufficient reaction kinetics at extreme dilution within aqueous environments, and could be controlled by a bioorthogonal stimulus that induces an on-demand switch from reversibility to irreversibility.

Herein, we report *click’n lock* as a principle for the chemoselective modification of biomolecules. We show that the combination of click reactions, de-/trans-click reactions and locking of chemical entities could provide maximum control over biomolecular architectures while still respecting the stringent criteria of click reactions including the absence of base or other additives. We exemplify the principle of *click’n lock* by employing the tetrazine–thiol exchange (TeTEx) as a click reaction. TeTEx involves the displacement of a methyl thiol moiety from a non-symmetric tetrazine selectively by thiol nucleophiles by means of a nucleophilic aromatic substitution (S_N_Ar). Notably, TeTEx is strongly accelerated in buffered aqueous media and allows quantitative conversion (98%) even at micromolar concentrations. We show that the reaction is reversible upon exposure to another thiol substrate, thus offering applications as a de-/trans-click reaction. In contrast to existing reversible click approaches, TeTEx can be locked by addition of a bioorthogonal stimulus in form of dienophiles providing maximal control over desired products with temporal resolution.^17,18^ Utilizing non-symmetric tetrazines allows installation of a functionality by click chemistry, which in turn can be released upon a de-/trans-click reaction. Alternatively, the conjugate can be locked, providing high tunability to the system. Finally, we envision that TeTEx opens new avenues in the field of click and conjugation chemistries because of its traceless and efficient nature.

## Results and discussion

### TeTEx: a reversible click reaction

Thiols are attractive substrates for click and de-/trans-click strategies of a variety of targets because of their presence in biomolecules, in the form of cysteines, alongside their high reactivity towards electrophiles. While numerous strategies are reported to selectively modify cysteines in complex biomolecules, only a few methods exist that are in principle reversible.^19–26^ In order to provide a single reaction system that not only combines both prospects, irreversible click chemistry and reversible de-/trans-click chemistry, but also is applicable to biomolecules, we turned our attention to S_N_Ar chemistries of tetrazines. While biomolecules have been selectively modified with tetrazines *via* S_N_Ar chemistry, once installed no substrate-induced reversibility of these reactions was reported.^27–32^ Interestingly, recent reports describe reversible S_N_Ar of 3,6-heteroatom bearing tetrazines, but indeed these systems require typically strong basic conditions and do not proceed at dilute aqueous conditions under click-like conditions.^17,18,33^ While reactivity of symmetric 3,6-heteroatom tetrazines was observed with hydrogen sulfide at dilute aqueous conditions, no reaction was observed with cysteines or other relevant thiol bearing biomolecules under such conditions.^33^ We hypothesized that a more reactive system would be obtained by making the tetrazine scaffold more electron deficient, thus enabling chemoselective click reactions and de-/trans-click reactions of biomolecules under dilute aqueous conditions.^34^ For this purpose, we turned our attention to non-symmetric tetrazines bearing a single sulfide residue.

**Fig. 2:**
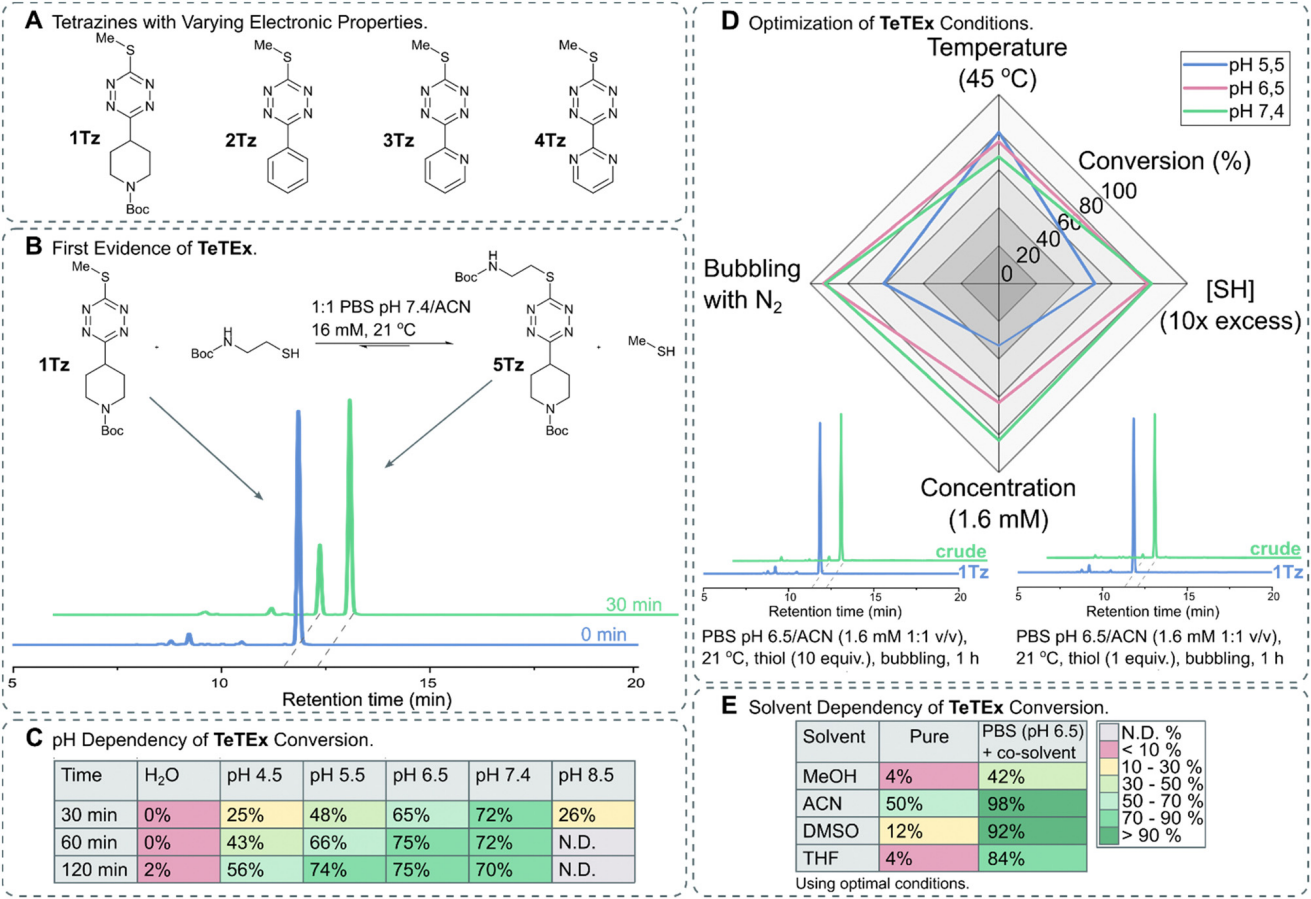
(A) Structures of 3-thiomethyl tetrazines 1Tz–4Tz with varying electronic properties. (B) TeTEx between 1Tz (16 Mm) and 2-(Boc-amino)ethanethiol (5 equiv.) in a PBS (pH 6.5)/ACN mixture (1 : 1 v/v) at 21 °C. Conversions were determined *via* RP-HPLC. Conversion of 70% was determined by RP-HPLC after 30 min. (C) Effect of pH on the conversion of TeTEx between 1Tz (1 equiv.) and 2-(Boc-amino)ethanethiol (5 equiv.). The reactions were performed in a 1 : 1 mixture of ACN with MilliQ water, citric acid buffer (pH 4.5 or 5.5), PBS buffer (pH 6.5 or 7.4), or sodium borate buffer (pH 8.5) (16 mM, 21 °C). Only at pH 8.5 tetrazine decomposition was observed, thus the conversion of the reaction could not be determined. (D) Effect of (i) increase in temperature to 45 °C, (ii) increase in thiol concentration [SH] to 10 equiv., (iii) decrease in tetrazine concentration to 1.6 mM and (iv) bubbling the solution through with N_2_. The conversions are shown in Table S2 (ESI† 5.3). Two representative RP-HPLC traces with quantitative yields (>97%) after using the determined optimal conditions. (E) TeTEx conversions between 1Tz (1 equiv.) and 2-(Boc-amino)ethanethiol (1 equiv.) while employing pure organic solvents at 21 °C, or mixtures of PBS (pH 6.5) and organic co-solvent (1.6 mM).

The synthesis of non-symmetric functional 1,2,4,5-tetrazines bearing a single methyl thiol moiety was accomplished using the recently reported protocol by the group of Fox.^35^ For this purpose, 3-oxetanemethanol derived esters were formed from a range of commercially available carboxylic acids bearing *N*-Boc-protected-piperidinyl, benzyl, pyridyl and pyrimidyl functionalities. In brief, oxetane esters were transformed into orthoesters upon treatment with BF_3_·OEt_2_ and subsequently condensed with methyl thiocarbohydrazide. The obtained 1,4-dihydro-1,2,4,5-tetrazines were oxidized yielding a range of 3-thiomethyl tetrazines with varying electronic properties (Fig. 2A).

To confirm our hypothesis that mono-thioether bearing 3,6-tetrazines can undergo S_N_Ar with thiol nucleophiles, we incubated 1Tz with 2-(Boc-amino)ethanethiol in PBS (pH 7.4) with acetonitrile as co-solvent and monitored the conversion of thiol displacement. After 30 minutes, 70% conversion was determined *via* RP-HPLC (Fig. 2B) confirming not only our hypothesis that thiols can act as both, nucleophiles and leaving groups, on non-symmetric tetrazines but also suggesting that this tetrazine–thiol exchange occurs under aqueous conditions in the absence of base or other additives. To confirm the product, 5Tz was isolated and its structure was confirmed *via* NMR and HRMS analysis (ESI† 4.1). Encouraged by these results, we aimed to identify the optimal conditions for TeTEx. Since thiol reactivity is pH dependent, different buffered media with pH values ranging from 4.5 to 8.5 were screened alongside with non-buffered organic solvent mixtures. It was determined that higher pH values resulted in higher conversions (Fig. 2C), while pH 8.5 was deemed incompatible due to tetrazine decomposition (Fig. S7 in ESI† 5.2). Additionally, the non-buffered system performed poorly even after 24 hours.

By increasing the amount of thiol substrate, we decreased the competition with the methyl thiol, thus improving the efficiency of the reaction. On the other hand, we hypothesized that increased temperature removes the gas side-product from the reaction mixture also improving the conversion of the conjugation.^15^

With these observations in mind, we aimed to shift the equilibrium to the product by removing the methyl thiol either by increasing the temperature or by bubbling the solution through with N_2_. Furthermore, to work in concentrations relevant for most click-like reactions employed for synthetically tailoring of biomolecules, we investigated the TeTEx conversion at a 10-fold decrease in tetrazine concentration.^36–38^ RP-HPLC analysis revealed that TeTEx at pH 6.5 performed well using these conditions, while the reaction was slower at pH 5.5 (Fig. 2D). Finally, quantitative yields (98%) were obtained by bubbling the solution through with N_2_, when employing equimolar amounts of thiol and tetrazine 1Tz (Fig. 2D), even at 200 μM concentrations (ESI† 5.5.3). Interestingly, thiol displacement was still inefficient in pure polar solvents like MeOH, DMSO, and THF even when saturated with inorganic salts (Table S3 in ESI† 5.3), suggesting that TeTEx is accelerated in aqueous buffered media (Fig. 2E). This intriguing acceleration of reactivity is currently being investigated.

**Fig. 3:**
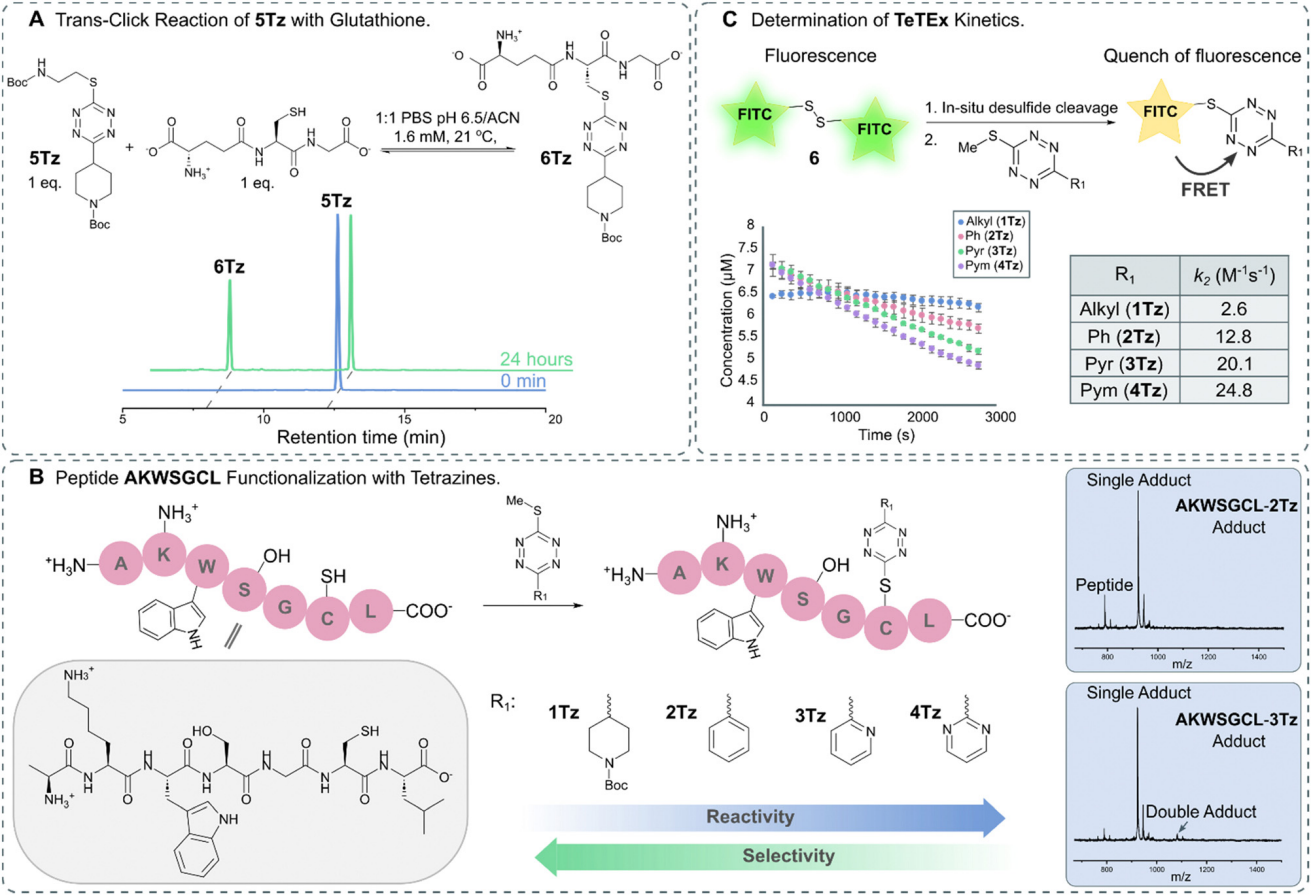
(A) HPLC trace of trans-click reaction between 5Tz and glutathione leading to 39% conversion after 24 hours. (B) Functionalization of peptide AKWSGCL with tetrazines displaying varying electronic propertied (R_1_ = Alkyl, 1Tz; R_1_ = Ph, 2Tz; R_1_ = Pyr, 3Tz; R_1_ = Pym, 4Tz). MALDI-TOF traces show that the chemoselectivity for thiols decreases with increasing electron deficiency (Fig. S20–S24 in ESI† 6.3). (C) Schematic representation of the kinetic assay in which the fluorescence is quenched upon reaction between FITC-cystamine 6 and 1Tz–4Tz. The kinetic assay was performed in triplicate.

For broadening the utility of TeTEx as a mild and traceless conjugation method in the context of late-stage tailoring of biomolecules, we investigated the influence of a reducing agent, namely tris(2-carboxyethyl)phosphine hydrochloride (TCEP). TCEP is known to reduce tetrazines to the corresponding dihydro tetrazines.^31^ Tetrazine 1Tz showed only little reduction in the presence of TCEP after 24 hours, which could be reversed by simply prolonged exposure to air (Fig. S13 and S14 in ESI† 6.1). Finally, the reversibility of the reaction was investigated by incubating the obtained 5Tz conjugate with equimolar amounts of glutathione and TCEP. After 2 hours, 12% conversion to the 6Tz was observed (Fig. 3A and Fig. S25, S26 in ESI† 7), proving that our system possesses trans-click potential. While we would like to emphasize that the current system is limited to an excess of reactant when full trans-click conversion is required, we believe that the trans-click capability should still be applicable to a variety of applications including affinity purification assays.

After gaining a fundamental understanding of TeTEx reactivity, we set out an investigation to determine the scope of TeTEx. We demonstrated that the nucleophilicity of the thiol substrates and stability of the acquired conjugate play a crucial role. Reaction with the sterically hindered *tert*-butyl thiol resulted in negligible conversion and reaction with thioacetic acid yielded a thioester prone to hydrolysis (Fig. S17 and S18 in ESI† 6.2). Next, we hypothesized that the substituents of the tetrazine determine the efficiency and selectivity of TeTEx. For this purpose, we investigated the effects of the tetrazine substitution patterns by reacting tetrazines displaying varying electronic properties (R_1_ = Alkyl, 1Tz; R_1_ = Ph, 2Tz; R_1_ = Pyr, 3Tz; R_1_ = Pym, 4Tz) with peptide AKWSGCL at equimolar concentration (*c* = 500 μM) (Fig. 3B). We observed for tetrazines with increasing electron-deficiency an increase in reactivity of the tetrazine towards substitution (Fig. S20–S24 in ESI† 6.3). Additionally, the more reactive tetrazines bearing pyridinyl and pyrimidyl functionalities lead to negligible and minor amounts of double-addition product, respectively (Fig. 3B). Our data suggests that the pyridinyl functionality provides an excellent balance between reactivity and chemoselectivity since peptide AKWSGCL displayed several reactivity centers (Cys, Lys, Ser, Trp and C-/N-terminus).

Tetrazines are heterocycles that quench fluorescence *via* Förster resonance energy transfer (FRET).^39^ For determining the second order rate constants *k*_2_ of TeTEx, FITC-cystamine 6 (6–7 μM) and tetrazines 1Tz–4Tz (1 equiv.) were incubated together and the decay in fluorescence (*λ*_em_ = 519 nm) was monitored over time. No decay in fluorescence was observed in the absence of tetrazines, while the fluorescence decreased after 1 hour in the presence of tetrazines 1Tz–4Tz. The second order rate constants *k*_2_ of 1Tz–4Tz were determined to be 2.6 M^−1^ s^−1^, 12.8 M^−1^ s^−1^, 20.1 M^−1^ s^−1^ and 24.8 M^−1^ s^−1^, respectively (Fig. 3C). These results align with the reactivity that was observed against peptide AKWSGCL. The reaction rates of TeTEx are significantly higher than many reported click reactions including SPAAC (for an overview of cysteine modifications see ESI† 9).^40,41^

### *Click’n lock*: chemically locking of reactions induces irreversibility

**Fig. 4:**
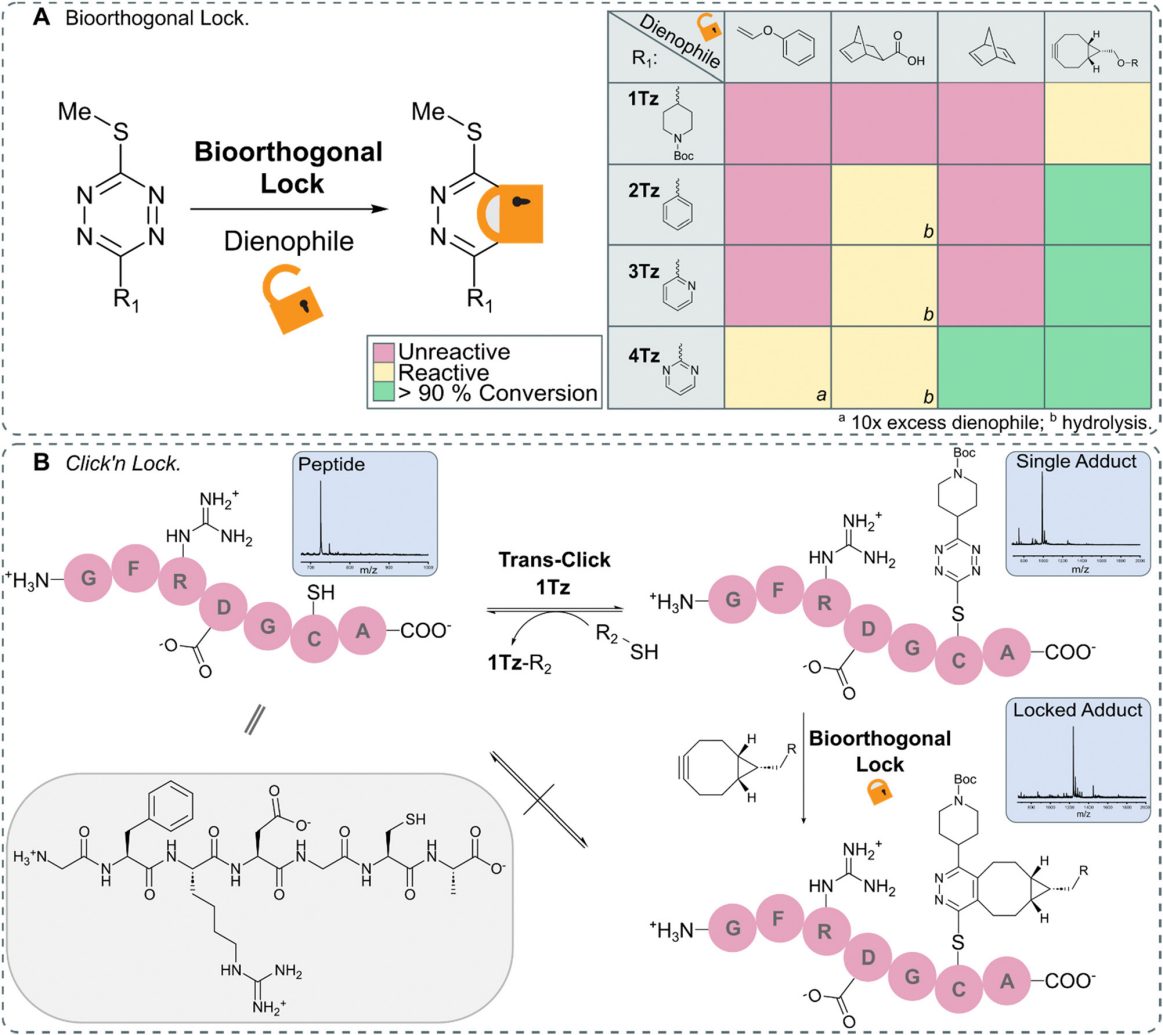
(A) Schematic representation of lock *via* IEDDA (left) and overview of the reactivity between 1Tz–4Tz and dienophiles (right). 1Tz–4Tz (500 μMM) and dienophiles (1.5 mM) in PBS (pH 6.5)/ACN (1 : 1 v/v) stirred at 37 °C for 24 hours; the reactions were monitored *via* LC-MS. (B) Peptide GFRDGCA (*c* = 500 μM) was decorated with 1Tz (*c* = 500 μM) using TeTEx. The adduct could either be de-clicked *via* addition of DTT (5 equiv.) or locked *via* addition of BCN (5 equiv.). Once locked, the adduct could no longer be de-clicked (Fig. S38–S47 in ESI† 10.3).

After identifying TeTEx as a powerful click reaction for the modification of biomolecules, susceptive of de-/trans-click chemistry, we turned our attention to providing a strategy that allows bioorthogonal locking of obtained products. Inducing irreversibility on-demand to an otherwise reversible reaction system, offers scientists great control over molecular processes and structures. For fulfilling the criteria of a fully orthogonal reaction system, we sought that the locking reaction should occur at sub-millimolar concentration without the need of elevated temperatures. To accomplish this aim, inspired by the group of Carrillo, we took advantage of the inherent reactivity of tetrazines towards IEDDA.^17,18^ We began our investigation by employing *exo*-5-norbornenecarboxylic acid as a dienophile due to its commercial availability, strain promoted reactivity and overall chemical stability (Fig. 4).^10^ While *exo*-5-norbornenecarboxylic acid reacted with 2Tz, 3Tz and 4Tz within buffered aqueous media, the 1,2-dihydropyridazine product was susceptible to hydrolysis resulting in the loss of the thiol cargo (ESI† 10.1). This observation led us to hypothesize that a dienophile which upon cycloaddition immediately provides the aromatic pyridazine would result in a stable locked structure. Phenyl vinyl ethers (PVEs) can undergo click-to-release reactions with tetrazines resulting in the aromatic conjugate.^42–44^ However, cycloaddition with PVE was only observed for the most activated tetrazine, 4Tz, when the dienophile was used in excess (Fig. S32 in ESI† 10.2). While no hydrolysis was observed when utilizing PVE, the IEDDA cycloaddition proceeded with unsatisfactory rates.

Despite the high ring-strain of norbornadiene, only cycloaddition with the activated 4Tz was observed. After a subsequent retro Diels–Alder reaction, the pyridazine product and the volatile by-product, cyclopentadiene were obtained and confirmed the hypothesis that an aromatic pyridazine must be obtained in order to provide a stabilized lock.^45^ Highly strained alkynes including bicyclononyne (BCN), are another class of dienophiles that provide the aromatic pyridazine upon IEDDA reaction and therefore would enable orthogonal locking of bioconjugates with high efficiency. While BCN displays some undesirable characteristics as it is a sterically demanding moiety that introduces a synthetically large overhead, it rapidly reacted with all tetrazines (Fig. S34–S37 in ESI† 10.2).

In order to demonstrate the full potential of TeTEx we decorated a small peptide GFRDGCA at equimolar concentration (*c* = 500 μM) in PBS (pH 6.5) with 1Tz and acetonitrile as co-solvent. Once the conjugation was complete, we incubated the construct with dithiothreitol (DTT) (5 equiv.). Full conversion was observed back to the free peptide indicating efficient de-click reaction. Alternatively, the peptide-1Tz conjugate was reacted with BCN (5 equiv.) to lock the stable pyridazinyl structure. This time, addition of DTT did not allow the de-click to proceed. The stability of the pyridazinyl product was also confirmed when 2-(Boc-amino)ethanethiol was used during TeTEx. After locking 5Tz with BCN, the resulting pyridazinyl product was stable after 4 days at 37 °C and subsequently 3 days in the presence of glutathione (ESI† 10.4). These findings demonstrate that TeTEx can be used for tunable conjugation purposes depending on the application at hand.

## Conclusions

Click chemistry involving thiols is often employed for the late-stage modification of complex structures. While there are numerous chemoselective click chemistries reported that enable precision synthesis at even extreme aqueous dilution, substrate-induced reversible reactions are scarce and often limited by their need for organic solvents or additional reagents. Herein, we report the use of the rapid exchange between non-symmetric tetrazines and thiols (TeTEx) for the reversible functionalization of biomolecules. TeTEx proceeds with quantitative conversion (98%) even at micromolar concentration within aqueous environments while still employing mild conditions such as equimolar concentrations of reactants and ambient temperature. Notably, TeTEx can be reversed by employing equimolar amounts of substrates within buffered aqueous environments, thus allowing new opportunities as chemoselective de-/trans-click reaction. The inherent reactivity of tetrazines towards IEDDA allows to stabilize the clicked structure, switching from a reversible to irreversible system (*click’n lock*). We believe that TeTEx will be a powerful addition to the chemical toolbox of click reactions because of its traceless nature and high chemoselectivity, not only in the field of chemical biology, but also for material science and nanomedicine.

## Author contributions

K. N. conceived and supervised the project. K. G. designed and conducted experiments. K. G. and D. D. carried out small molecule synthesis. D. G. carried out peptide synthesis. K. G. conduct kinetics measurements, conjugation assays, optimisation studies and stability assays. K. N. and K. G. wrote the manuscript.

## Conflicts of interest

There are no conflicts to declare.

## Supplementary Material

CB-004-D3CB00062A-s001
