# Supplementary material for: Click’n lock: rapid exchange between unsymmetric tetrazines and thiols for reversible, chemoselective functionalisation of biomolecules with on-demand bioorthogonal locking
Source: RSC Chem Biol. 2023 Jul 20;4(9):685–91. doi: 10.1039/d3cb00062a (PMC10467616; doi:10.1039/d3cb00062a)
Supplement: CB-004-D3CB00062A-s001 [file CB-004-D3CB00062A-s001.pdf]

Electronic Supplementary Material (ESI) for RSC Chemical Biology.
This journal is © The Royal Society of Chemistry 2023

 DOI: 10.1039/x0xx00000x

# *Click'n lock: Rapid exchange between unsymmetric tetrazines and thiols for reversible, chemoselective functionalisation of biomolecules with on-demand bioorthogonal locking*

Katerina Gavriel[a], Dustin C. A. van Doeselaar[a], Daniëlle W. T. Geers[a] and Kevin Neumann*[a]

[a] Systems Chemistry Department, Institute for Molecules and Materials, Radboud University Nijmegen, Heyendaalseweg 135, 6525 AJ Nijmegen, The Netherlands

kevin.neumann@ru.nl

**Table of Contents**

Experimental Procedures....4
1. Materials....4
2. Instrumentation....4
3. Experimental protocols....5
3.1. Methyl thiocarbohydrazide hydrogen iodide salt 1....5
3.2. General protocol 1 for oxetane ester synthesislock....5
3.2.1. Oxetane ester **2**....5
3.2.2. Oxetane ester **3**....5
3.3. General protocol 2 for oxetane ester synthesis....6
3.3.1. Oxetane ester **4**....6
3.3.2. Oxetane ester **5**....6
3.4. General protocol for the synthesis of non-symmetric tetrazines bearing a methyl thiol moiety ...6
3.4.1. N-Boc protected piperidinyl-functionalized tetrazine (**1Tz**)....7
3.4.2. Benzyl-functionalized tetrazine (**2Tz**)....7
3.4.3. Pyridyl-functionalized tetrazine (**3Tz**)....7
3.4.4. Pyrimidyl-functionalized tetrazine (**4Tz**)....7
3.5. FITC-cystamine 6....8
3.6. General protocol for peptide synthesis....8
3.6.1. Alanine-Lysine-Tryptophan-Serine-Glycine-Cysteine-Leucine (**AKWSGCL**)....8
4. General protocol for tetrazine-thiol exchange (TeTEx)....10
4.1. tert-butyl 4-(6-((2-((tert-butoxycarbonyl)amino)ethyl)thio)-1,2,4,5-tetrazin-3-yl)piperidine-1-carboxylate (**5Tz**)....10
Results and Discussion....11
5. Optimization of TeTEx conditions with 1Tz and 2-(Boc-amino) ethanethiol....11
5.1. Initial Standard Conditions....11
5.2. Representative selection of HPLC traces showing the pH dependance on TeTEx conversion ......11
5.3. Determination of optimal conditions....14
5.4. Postulated reaction mechanism of TeTEx with 3-thiomethyl tetrazines....15
5.5. Representative selection of HPLC traces during the optimization of TeTEx conditions....15
5.5.1. HPLC trace of **TeTEx** at the determined optimal conditions with excess thiol.....15
5.5.2. HPLC trace of **TeTEx** at the determined optimal conditions with stoichiometric amounts of thiol.....16
5.5.3. HPLC trace of **TeTEx** at the determined optimal conditions at 200 μM concentration.....16
5.5.4. Tetrazine decomposition at pH 7.4 when excess thiol or increased temperature was employed. ....17
6. Scope determination for TeTEx....17
6.1. Compatibility of 1Tz with TCEP....17
6.2. Dependance of thiol nucleophilicity in the efficiency of TeTEx....18

DOI: 10.1039/x0xx00000x

6.2.1. Glutathione ....................19
6.2.2. tert-Butylthiol ....................20
6.2.3. Thioacetic acid ....................20
6.2.4. Thiophenol ....................21
6.3. Tetrazine electronic properties effect on TeTEx efficiency and selectivity ....................21
7. Investigation of trans-click reaction ....................23
8. Fluorescent assay for kinetic determination ....................24
9. Cysteine electrophiles ....................27
10. Investigation of Click'n Lock ....................27
10.1. Hydrolysis of 1,2-dihydropyridazine upon IEDDA ....................28
10.2. Representative selection of HPLC traces displaying lock via IEDDA ....................28
10.3. Click'n Lock demonstrated on peptide GFRDGCA ....................30
10.4. Click'n Lock demonstrated with small molecule thiol ....................35
11. NMR Spectra ....................38
References ....................48

 DOI: 10.1039/x0xx00000x

# Experimental Procedures

## 1. Materials

Reagents were obtained from Sigma Aldrich/Merck (Zwijndrecht, The Netherlands), Fluorochem BV (Amsterdam, The Netherlands) and TCI Europe (Zwijndrecht, Belgium). The reagents were used without purification unless otherwise stated. Solvents were obtained from VWR, Fisher, Acros Organic and Sigma Aldrich/Merck. Solvents were dried by passing over activated alumina columns in a MBraun MB SPS800 under a nitrogen atmosphere and stored under nitrogen. Reactions were carried under air unless stated otherwise. Air-sensitive reactions were carried out under atmosphere of nitrogen using Schlenk technique. Ultrapure Milli-Q water was obtained from QPOD Milli-Q system. Reactions and fractions from flash column chromatography were monitored by thin layer chromatography using glass TLC plates (Merck, TLC Silica gel 60 F254) and if necessary visualized by staining with potassium permanganate or ninhydrin solution. Column chromatography was performed on VWR SiO2 Type (40-63 mesh) using a forced flow of air at 0.5-1.0 bar.

## 2. Instrumentation

Nuclear Magnetic resonance (NMR) characterization was carried out on a Bruker AVANCE HD nanobay console with a 9.4 T Ascend magnet (400 MHz) and a Bruker AVANCE III console with a 11.7 T UltraShield Plus magnet (500 MHz) equipped with a Bruker Prodigy cryoprobe, in chloroform ($CDCl_3$) or DMSO-$d_6$. NMR spectra were recorded at 298 K unless otherwise stated. Tetramethylsilane (TMS, δ 0.00 ppm) was used as an internal standard for $^1$H NMR. Chemical shifts are reported in parts per million (ppm) and coupling constants are reported as *J* values in Hz. Peak assignment is based on 2D COSY, $^1$H–$^{13}$C HSQC, and $^1$H–$^{13}$C HMBC spectra. The splitting patterns are indicated as follows: s, singlet; br. s, broad singlet; d, doublet; t, triplet; m, multiplet. Mass spectra were measured with Bruker Microflex LRF Maldi-TOF system and JEOL AccuTOF CS JMST100CS. Electrospray liquid chromatography-mass spectrometry (LC-MS) was performed on a Single-Quad Thermo instrument equipped with a Thermo Scientific Accucore C18 (2.6 µm, 80 Å, 100 x 3 mm) column using 0.1% formic acid in ACN and in MilliQ as eluents and deferential refractive index or UV absorbance (254 nm). Reverse-phase high-performance liquid chromatography (RP-HPLC) was carried out on an Agilent AG1120 instrument equipped with a Phenomenex PRODIGY ODS (3) (5 µm, 100 Å, 250 x 4.60 mm) column using 0.1% formic acid in MilliQ and ACN (5-95% ACN in MilliQ over 25 min) as eluents and UV absorbance (215 and 254 nm). The peptides were purified using a Shimadzu LC-20 RP-HPLC equipped with a Phenomenex C18 column using 0.1% formic acid in ACN and in MilliQ as eluents and deferential refractive index or UV absorbance (254 nm). Up to 1 mL reactions were performed using a ThermoMixer C with SmartBlock, 14-285-562PM. Fluorescence measurements were performed on a Tecan Spark M10 plate reader using Greiner Bio-One black PS F-bottom 96 well microplates.

 DOI: 10.1039/x0xx00000x

## 3. Experimental protocols

### 3.1. Methyl thiocarbohydrazide hydrogen iodide salt 1

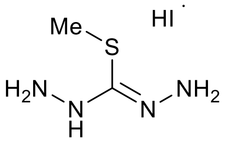


Compound **1** was prepared following a reported protocol.[1] A solution of thiocarbohydrazide (5.0 g, 47.1 mmol, 1 equiv.) and methyl iodide (3.2 mL, 51.8 mmol, 1.1 equiv.) in ethanol (0.3 M) was added in a dry round-bottom flask. The solution was refluxed at 80°C for 3 h, and cooled to room temperature. The reaction mixture was diluted with heptane and cooled to –20°C overnight. The resulting solid was collected via filtration, washed with a cold ethanol/heptane (1:1 v/v) mixture and dried under reduced pressure. The product was obtained as a white solid (8.9 g, 76% yield) and the spectroscopic data were in agreement with literature data. $^{1}$H NMR (400 MHz, DMSO) δ 9.62 (s, 1H), 5.15 (s, 2H), 3.33 (s, 1H), 2.39 (s, 3H). MS (ESI): calculated for $[C_2H_9N_4S]^+$: m/z 121.1, found: m/z 121.1.

### 3.2. General protocol 1 for oxetane ester synthesislock

Oxetane ester synthesis was performed following a reported protocol.[1] 3-methyl-3-oxetanemethanol (1.1 – 1.5 equiv.), EDC·HCl (1.2 – 1.5 equiv.) and DMAP (0.1 equiv.) were added in a dry flask under inert atmosphere. Anhydrous DCM or DMF was added to the flask and the solution was cooled to 0°C. Carboxylic acid (1 equiv.) was added and the solution was stirred under nitrogen at 0°C for 15 min and at room temperature overnight. The reaction mixture was diluted with DCM and washed with saturated sodium bicarbonate solution, water and brine. The organic layer was dried over sodium sulfate and concentrated under reduced pressure. The residue was purified by flash chromatography.

#### *3.2.1. Oxetane ester **2***

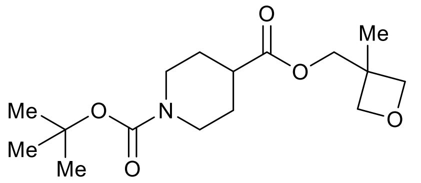


The general protocol 1 was followed using 1-Boc-piperidine-4-carboxylic acid (10.0 g, 43.6 mmol), 3-methyl-3-oxetanemethanol (4.8 mL, 48.0 mmol, 1.1 equiv.), EDC·HCl (10.0 g, 52.3 mmol, 1.2 equiv.), DMAP (0.5 g, 4.0 mmol, 0.1 equiv.) in DCM (0.3 M). A colorless oil (13.3 g, 97% yield) was obtained after flash chromatography (10 – 50% EtOAc in Heptane). $^{1}$H NMR (400 MHz, $CDCl_3$) δ 4.51 (d, *J* = 6.0 Hz, 2H), 4.39 (d, *J* = 6.0 Hz, 2H), 4.17 (s, 2H), 4.04 (d, *J* = 13.3 Hz, 2H), 2.84 (t, *J* = 12.4 Hz, 2H), 2.52 (tt, *J* = 11.1, 3.9 Hz, 1H), 1.98 – 1.83 (m, 2H), 1.72 – 1.58 (m, 2H), 1.46 (s, 9H), 1.33 (s, 3H). $^{13}$C NMR (101 MHz, $CDCl_3$) δ 174.7, 154.8, 79.8, 79.6, 68.7, 41.3, 39.3, 28.6, 28.1, 21.3. HRMS (ESI): calculated for $[C_{16}H_{27}NO_5Na]^+$: m/z 336.1787, found: m/z 336.1775.

#### *3.2.2. Oxetane ester **3***

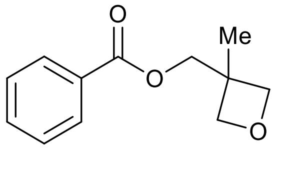

The general protocol 1 was followed using benzoic acid (1.0 g, 8.2 mmol), 3-methyl-3-oxetanemethanol (0.9 mL, 9.0 mmol, 1.1 equiv.), EDC·HCl (1.9 g, 9.8 mmol, 1.2 equiv.), DMAP (0.1 g, 0.8 mmol, 0.1 equiv.) in DCM (0.3 M). A colorless oil (1.2 g, 73% yield) was obtained after flash chromatography (10% EtOAc in Heptane). $^{1}$H NMR (400 MHz, $CDCl_3$) δ 8.17 – 7.93 (m, 2H), 7.67 – 7.51 (m, 1H), 7.49 – 7.40 (m, 2H), 4.64 (d, *J* = 6.0 Hz, 2H), 4.45 (d, *J* = 6.0 Hz, 2H), 4.39 (s, 2H), 1.42 (s, 3H). $^{13}$C NMR (101 MHz, $CDCl_3$) δ 166.5, 133.2, 129.9, 129.6, 128.5, 79.6, 69.0, 39.3, 21.3. HRMS (ESI): calculated for $[C_{12}H_{15}O_3]^+$: m/z 207.10212, found: m/z 207.10149.

## 3.3. General protocol 2 for oxetane ester synthesis

Carboxylic acid, EDC·HCl and DMAP or EDC·HCl, HOBt and DIPEA were added in a dry flask under inert atmosphere. Anhydrous DMF was added to the flask and the solution was stirred for 15 min at 0°C. 3-methyl-3-oxetane methanol was added to the reaction mixture and stirred overnight. The reaction mixture was diluted with water and washed with DCM and EtOAc. The combined organic layer was dried over sodium sulfate and concentrated under reduced pressure. The residue was purified by flash chromatography.

### *3.3.1. Oxetane ester **4***

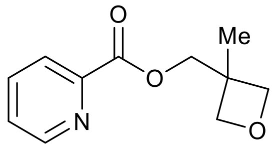


The general protocol 2 was followed using picolinic acid (250.0 mg, 2.0 mmol), 3-methyl-3-oxetanemethanol (0.3 mL, 3.0 mmol, 1.5 equiv.), EDC·HCl (584.0 g, 3.1 mmol, 1.5 equiv.), DMAP (24.8 mg, 0.2 mmol, 0.1 equiv.) in DMF (0.2 M). A white solid (124.7 mg, 30% yield) was obtained after flash chromatography (67% EtOAc in Heptane). $^{1}$H NMR (400 MHz, $CDCl_3$) δ 8.82 (ddd, *J* = 4.7, 1.8, 0.9 Hz, 1H), 8.15 (dt, *J* = 7.8, 1.1 Hz, 1H), 7.88 (td, *J* = 7.8, 1.8 Hz, 1H), 7.52 (ddd, *J* = 7.6, 4.7, 1.2 Hz, 1H), 4.69 (d, *J* = 6.0 Hz, 2H), 4.54 (s, 2H), 4.49 (d, *J* = 6.0 Hz, 2H), 1.47 (s, 3H). $^{13}$C NMR (126 MHz, $CDCl_3$) δ 165.1, 161.8, 150.1, 147.8, 137.0, 127.0, 125.2, 79.6, 77.0, 69.8, 39.5, 29.7, 21.2. HRMS (ESI): calculated for $[C_{11}H_{13}NO_3Na]^+$: m/z 230.0793, found: m/z 230.0769.

### *3.3.2. Oxetane ester **5***

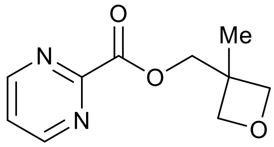


The general protocol 2 was followed using 5-hydroxy picolinic acid (491.3 mg, 3.5 mmol), 3-methyl-3-oxetane methanol (0.8 mL, 8.1 mmol, 2.3 equiv.), EDC·HCl (1776.4 mg, 9.3 mmol, 2.6 equiv.), HOBt (1234.0 mg, 9.1 mmol, 2.6 equiv.) and DIPEA (1.4 mL, 8.1 mmol, 2.3 equiv.) in DMF (0.2 M). A white solid (279.1 mg, 35% yield) was obtained after flash chromatography (100% EtOAc). $^{1}$H NMR (400 MHz, $CDCl_3$) δ 8.96 (d, *J* = 4.9 Hz, 2H), 7.51 (t, *J* = 4.9 Hz, 1H), 4.67 (d, *J* = 6.1 Hz, 2H), 4.61 (s, 2H), 4.47 (d, *J* = 6.1 Hz, 2H), 1.47 (s, 3H). $^{13}$C NMR (101 MHz, $CDCl_3$) δ 163.7, 157.9, 156.7, 123.2, 79.6, 77.1, 70.6, 39.5, 21.2. HRMS (ESI): calculated for $[C_{10}H_{12}N_2O_3Na]^+$: m/z 231.0746, found: m/z 231.0742.

## 3.4. General protocol for the synthesis of non-symmetric tetrazines bearing a methyl thiol moiety

Tetrazine synthesis was performed following a reported protocol.[1] A solution of oxetane ester (1.2 equiv.) in anhydrous DCM (1.0 M) was added in a dry round-bottom flask. The solution was cooled to –12°C and boron trifluoride etherate (1.2 equiv.) was added dropwise. The mixture was stirred for 4 – 6 h under nitrogen while cooled at –12°C. Once the reaction was complete, the reaction mixture was quenched with pyridine (2.5 equiv.), and a solution of methyl thiocarbohydrazide iodide salt **1** (1 equiv.) in DMF (1.0 M) was added. The temperature of the mixture was allowed to slowly reach 80 °C while nitrogen flow was applied to the open flask in order to remove the DCM. The resulting mixture was allowed to stir for 30 min under nitrogen at 80°C. After cooling to room temperature, phenyliodine(III) diacetate (PIDA) (1 equiv.) was added to the flask and the mixture was allowed to stir for 30 min. The mixture was diluted with DCM and washed with saturated sodium

 DOI: 10.1039/x0xx00000x

bicarbonate, water and brine, dried over sodium sulfate and concentrated under reduced pressure. The residue was purified by flash chromatography.

### *3.4.1. N-Boc protected piperidinyl-functionalized tetrazine (**1Tz**)*

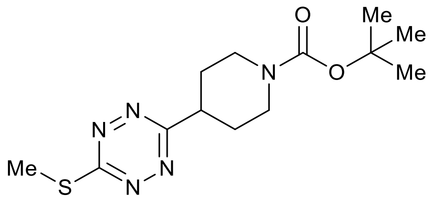

The general protocol was followed using oxetane ester **2** (13.0 g, 41.6 mmol), boron trifluoride etherate (5.2 mL, 41.6 mmol), pyridine (6.5 mL, 87.1 mmol), methyl thiocarbohydrazide iodide salt **1** (9.1 g, 35.1 mmol), PIDA (11.7 g, 35.1 mmol). A pink solid (4.2 g, 39% yield) was obtained after flash chromatography (10% EtOAc in Heptane). $^1$H NMR (400 MHz, $CDCl_3$) δ 4.27 (s, 2H), 3.39 (tt, *J* = 11.7, 3.8 Hz, 1H), 2.95 (t, *J* = 13.5 Hz, 2H), 2.74 (s, 3H), 2.10 (d, *J* = 13.1 Hz, 2H), 1.95 (dtd, *J* = 13.4, 11.9, 4.3 Hz, 2H), 1.48 (s, 9H). $^{13}$C NMR (101 MHz, $CDCl_3$) δ 176.0, 169.4, 154.9, 79.9, 41.5, 30.3, 28.6, 13.5. HRMS (ESI): calculated for $[C_{13}H_{21}N_5O_2SNa]^+$: m/z 334.1314, found: m/z 334.1328.

### *3.4.2. Benzyl-functionalized tetrazine (**2Tz**)*

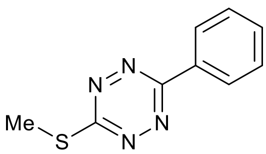

The general protocol was followed using oxetane ester **3** (1.3 g, 6.4 mmol), boron trifluoride etherate (0.8 mL, 6.4 mmol), pyridine (1.1 mL, 13.3 mmol), methyl thiocarbohydrazide iodide salt **1** (1.3 g, 5.3 mmol), PIDA (1.7 g, 4.2 mmol). A pink solid (0.3 g, 27% yield) was obtained after flash chromatography (10% EtOAc in Heptane). $^1$H NMR (400 MHz, $CDCl_3$) δ 8.56 – 8.41 (m, 2H), 7.69 – 7.47 (m, 3H), 2.80 (s, 3H). $^{13}$C NMR (101 MHz, $CDCl_3$) δ 175.4, 162.5, 132.4, 131.8, 129.4, 127.6, 13.6. MS (ESI): calculated for $[C_9H_9N_4S]^+$: m/z 205.1, found: m/z 205.2.

### *3.4.3. Pyridyl-functionalized tetrazine (**3Tz**)*

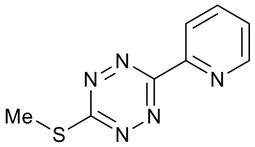

The general protocol was followed using oxetane ester **4** (103.6 mg, 0.5 mmol), boron trifluoride etherate (61.7 µL, 0.5 mmol), pyridine (100.7 µL, 1.3 mmol), methyl thiocarbohydrazide iodide salt **1** (103.4 mg, 0.4 mmol), PIDA (134.2 mg, 0.4 mmol). A pink solid (12.4 mg, 12% yield) was obtained after flash chromatography (67% EtOAc in Heptane). $^1$H NMR (500 MHz, $CDCl_3$) δ 8.95 (ddd, *J* = 4.8, 1.8, 0.9 Hz, 1H), 8.60 (dd, *J* = 7.9, 1.0 Hz, 1H), 7.98 (td, *J* = 7.8, 1.8 Hz, 1H), 7.55 (ddd, *J* = 7.6, 4.7, 1.2 Hz, 1H), 2.85 (s, 3H). $^{13}$C NMR (126 MHz, $CDCl_3$) δ 176.5, 150.8, 150.2, 137.4, 126.1, 123.4, 77.0, 13.5. MS (ESI): calculated for $[C_8H_8N_5S]^+$: m/z 206.0, found: m/z 206.1.

### *3.4.4. Pyrimidyl-functionalized tetrazine (**4Tz**)*

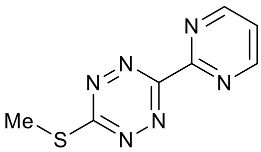

DOI: 10.1039/x0xx00000x

The general protocol was followed using oxetane ester **5** (107.1 mg, 0.5 mmol), boron trifluoride etherate (59.3 µL, 0.5 mmol), pyridine (96.7 µL, 1.2 mmol), methyl thiocarbohydrazide iodide salt **1** (99.3 mg, 0.4 mmol), PIDA (128.8 mg, 0.4 mmol). A pink solid (19.3 mg, 18% yield) was obtained after flash chromatography (100% EtOAc). $^{1}$H NMR (400 MHz, $CDCl_3$) δ 9.11 (d, *J* = 4.9 Hz, 2H), 7.57 (t, *J* = 4.9 Hz, 1H), 2.87 (s, 3H). $^{13}$C NMR (126 MHz, $CDCl_3$) δ 177.4, 161.3, 159.5, 158.4, 122.3, 77.0, 13.6. MS (ESI): calculated for $[C_7H_7N_6S]^+$: m/z 207.0, found: m/z 207.1.

### 3.5. FITC-cystamine 6

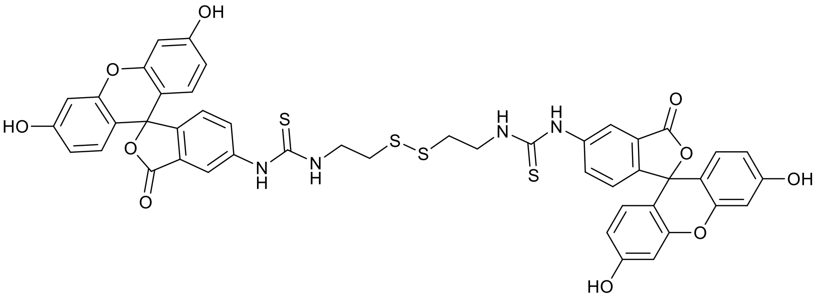


5-Isothiocyanato fluorescein (FITC) (182.0 mg, 0.5 mmol, 2.1 equiv.) and cystamine dihydrochloride (50.0 mg, 0.2 mmol, 1.0 equiv.) were dissolved in anhydrous DMF (30 mM). DIPEA (77.0 µL, 0.4 mmol, 2.0 equiv.) was added in the solution and the mixture was stirred at room temperature for 2 h. The reaction mixture was precipitated in DCM, and the orange solids (74.4 mg, 36%) were collected via high-speed centrifugation (4000 RPM for 5 min) and dried under reduced pressure. The compound was used without further purification. $^{1}$H NMR (400 MHz, DMSO) δ 10.18 (s, 4H), 8.24 (d, *J* = 2.0 Hz, 2H), 7.75 (d, *J* = 8.3 Hz, 2H), 7.18 (d, *J* = 8.3 Hz, 2H), 6.66 (d, *J* = 2.3 Hz, 4H), 6.63 – 6.48 (m, 8H), 3.85 (d, *J* = 8.5 Hz, 4H), 3.03 (t, *J* = 6.8 Hz, 4H), 2.89 (s, 2H), 2.73 (s, 2H). MS (ESI): calculated for $[C_{46}H_{35}N_4O_{10}S_4]^+$: m/z 931.1; found m/z 931.1.

### 3.6. General protocol for peptide synthesis

Peptides were synthesized on Wang resin by manual Fmoc-SPPS using HBTU activation procedure. Amino acid residues were coupled using a pre-activated solution of protected amino acid (0.3 M in DMF, 4.1 equiv.), HBTU (4 equiv.), and DIPEA. After 1 – 3 h, the resin was washed with DMF (x2), DCM (x2) and DMF (x2). Each amino acid coupling was performed twice. Upon complete amino acid coupling, the resin was capped using a DMF/DIPEA/acetic anhydride solution (10:2:1 v/v/v). The resin was washed and piperidine (20% in DMF) was added to the resin for 15 min. to remove the Fmoc protecting group. After chain assembly was completed, the peptide was deprotected and cleaved from the resin by treatment with a mixture of 4% $H_2O$, 4% TES and TFA. After 90 min., the peptide was precipitated in cold diethyl ether and centrifuged. The pellet of crude peptides was dissolved in ACN/$H_2O$ (1:1, v/v) and purified using preparative RP-HPLC.

#### *3.6.1. Alanine-Lysine-Tryptophan-Serine-Glycine-Cysteine-Leucine (**AKWSGCL**)*

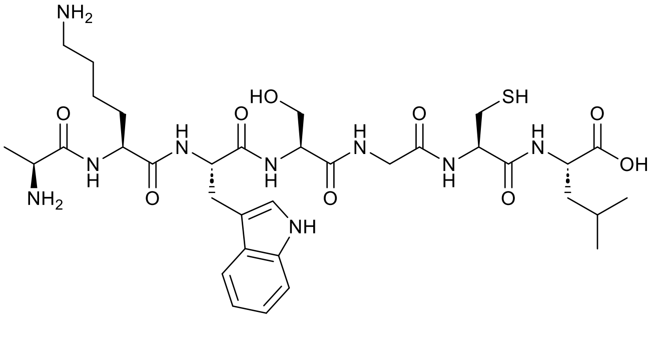


The crude peptide was purified using preparative HPLC (0 - 80% ACN in MilliQ over 20 min, 80 - 100% ACN in MilliQ over 2 min, 100% ACN in MilliQ for 3 min, 100 - 0% ACN in MilliQ over 3 min) (tr = 14.823 min). HRMS (ESI): calculated for $[C_{34}H_{54}N_9O_9S]^+$: m/z 764.3765, found: m/z 764.3762.

 DOI: 10.1039/x0xx00000x

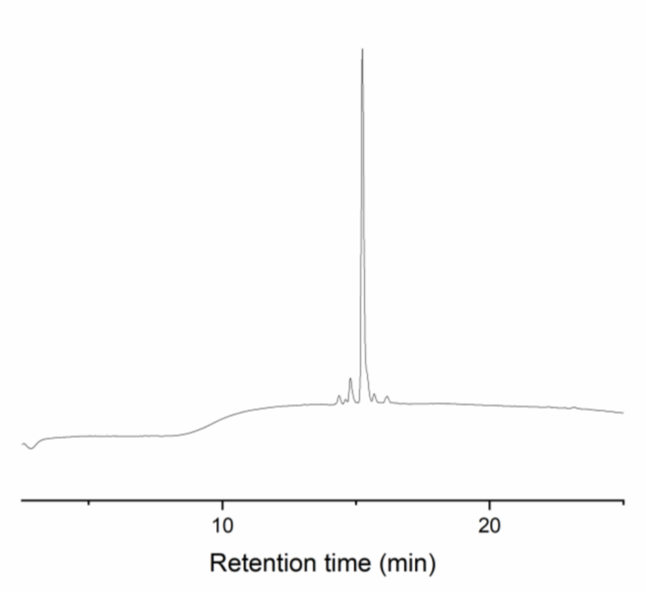


**Figure S1.** HPLC trace of peptide **AKWSGCL**. The peptide displays an 87% purity.

### 3.6.2. Glycine-Phenylalanine-Arginine-Aspartic Acid-Glycine-Cysteine-Alanine (**GFRDGCA**)

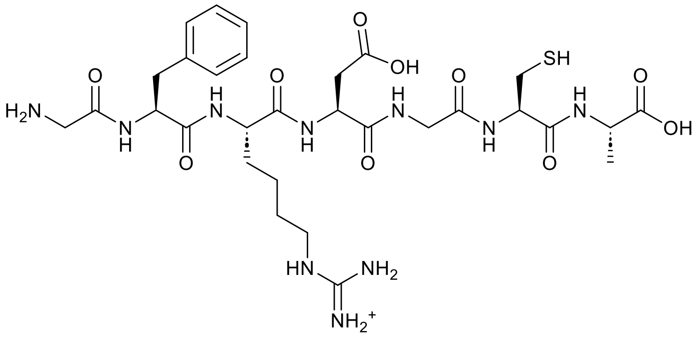


The crude peptide was purified using preparative HPLC (0 - 80% ACN in MilliQ over 15 min, 80 - 100% ACN in MilliQ over 1 min, 100% ACN in MilliQ for 3 min, 100 - 0% ACN in MilliQ over 2 min) (tr = 11.860 min). HRMS (ESI): calculated for $[C_{29}H_{45}N_{10}O_{10}S]^+$: m/z 725.3041, found: m/z 725.3067.

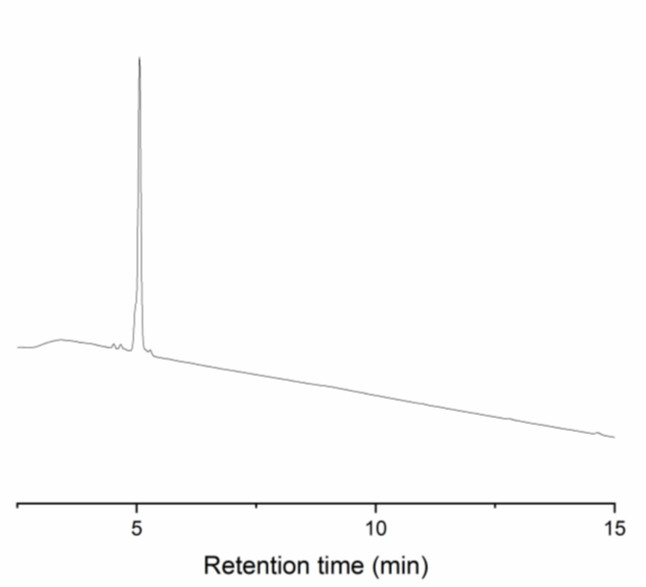


**Figure S2.** HPLC trace of peptide **GFRDGCA**. The peptide displays a 96% purity.

## 4. General protocol for tetrazine-thiol exchange (TeTEx)

Tetrazine was dissolved in a PBS (pH 6.5)/ACN solution. Thiol was added and the reaction was allowed to proceed at 21°C and 300 rpm while bubbling the solution through with $N_2$ for 0.5 – 2 hours. The conversion was monitored using RP-HPLC.

### *4.1. tert-butyl 4-(6-((2-((tert-butoxycarbonyl)amino)ethyl)thio)-1,2,4,5-tetrazin-3-yl)piperidine-1-carboxylate (**5Tz**)*

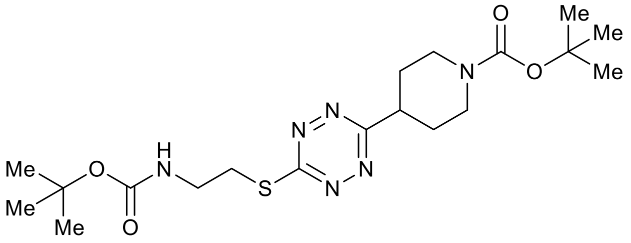


The general protocol was followed using a 1.6 mM solution of **1Tz** (0.5 mg, 1.6 µmol, 1 equiv.) in a PBS (pH 6.5)/ACN mixture (1:1 v/v) and 2-(Boc-amino) ethanethiol (0.3 µL, 1.6 µmol, 1 equiv.) for 1 hour. Upon completion, the aqueous solution was washed with DCM three times, dried over sodium sulfate and concentrated under reduced pressure. The residue was analysed without further purification. $^1$H NMR (400 MHz, $CDCl_3$) δ 4.90 (s, 1H), 3.54 (t, *J* = 6.2 Hz, 2H), 3.46 (t, *J* = 6.2 Hz, 2H), 3.38 (tt, *J* = 11.6, 3.8 Hz, 1H), 2.10 (d, *J* = 13.1 Hz, 2H), 1.94 (dtd, *J* = 13.3, 12.0, 4.3 Hz, 2H), 1.48 (s, 8H), 1.44 (s, 9H). $^{13}$C NMR (126 MHz, $CDCl_3$) δ 175.2, 169.6, 155.8, 154.7, 79.8, 41.3, 39.4, 30.7, 30.2, 28.5, 28.4. HRMS (ESI): calculated for $[C_{19}H_{32}N_6O_4SNa]$ +: m/z 463.2103, found: m/z 463.2079.

 DOI: 10.1039/x0xx00000x

# Results and Discussion

## 5. Optimization of TeTEx conditions with 1Tz and 2-(Boc-amino) ethanethiol

### 5.1. Initial Standard Conditions

**1Tz** (5 mg, 16.1 µmol, 1 equiv.) was dissolved in a 1 mL buffer/ACN solution (16 mM, 1:1 v/v). 2-(Boc-amino) ethanethiol (14 µL, 80.5 µmol, 5 equiv.) was added and the reaction was allowed to proceed at 21°C and 300 rpm for up to 24 h. The conversion was monitored using RP-HPLC.

**Table S1.** Buffers displaying different pH values used for the optimization of the reaction conditions.

| Buffers | pH |
|---|---|
| Citric Acid Buffer | 4.5 |
| Citric Acid Buffer | 5.5 |
| PBS Buffer | 6.5 |
| PBS Buffer | 7.4 |
| Sodium Borate Buffer | 8.5 |

### 5.2. Representative selection of HPLC traces showing the pH dependance on TeTEx conversion

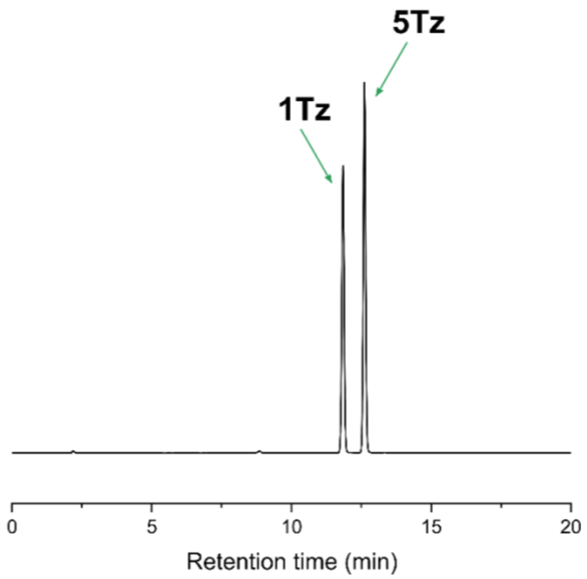


**Figure S3.** HPLC trace **TeTEx** between **1Tz** and 2-(Boc-amino) ethanethiol at pH 4.5 after 2 h reaction time. 56% conversion is determined from the area under the curve.

 DOI: 10.1039/x0xx00000x

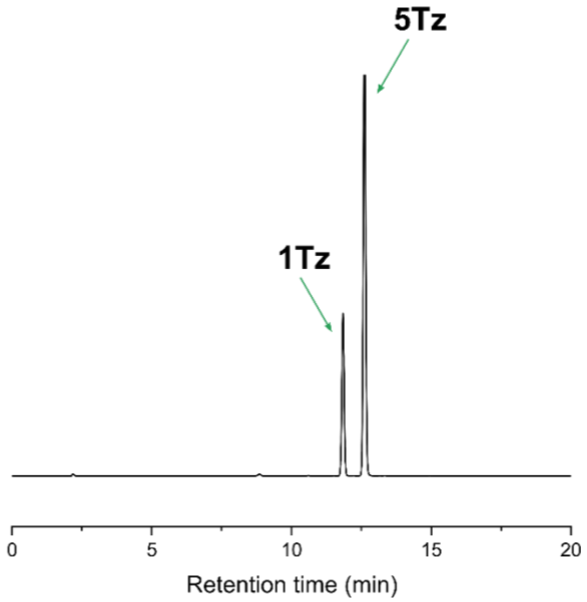


**Figure S4.** HPLC trace **TeTEx** between **1Tz** and 2-(Boc-amino) ethanethiol at pH 5.5 after 2 h reaction time. 74% conversion is determined from the area under the curve.

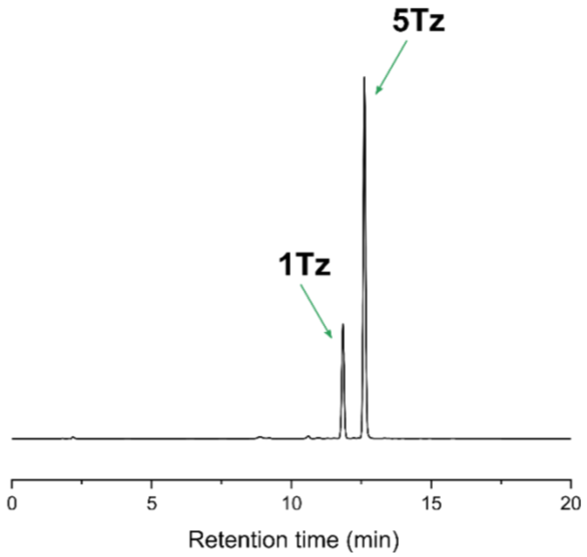


**Figure S5.** HPLC trace **TeTEx** between **1Tz** and 2-(Boc-amino) ethanethiol at pH 6.5 after 2 h reaction time. 75% conversion is determined from the area under the curve.

DOI: 10.1039/x0xx00000x

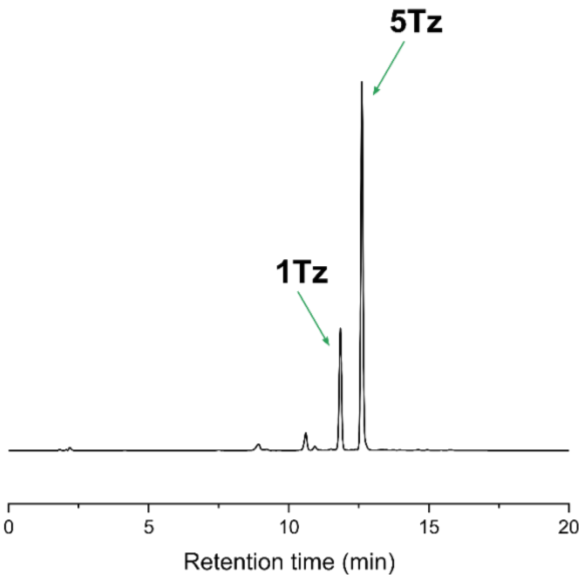


**Figure S6.** HPLC trace **TeTEx** between **1Tz** and 2-(Boc-amino) ethanethiol at pH 7.4 after 2 h reaction time. The appearance of new peaks below tr = 11.5 indicate decomposition of the tetrazine. 70% conversion is determined from the area under the curve.

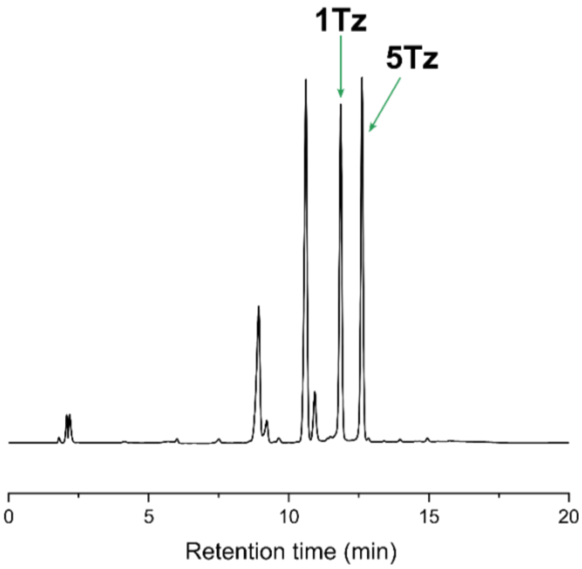


**Figure S7.** HPLC trace **TeTEx** between **1Tz** and 2-(Boc-amino) ethanethiol at pH 8.5 after 30 min reaction time. The appearance of new peaks below tr = 11.5 indicate decomposition of the tetrazine. 26% conversion is determined from the area under the curve.

DOI: 10.1039/x0xx00000x

## 5.3. Determination of optimal conditions

The initial standard protocol was followed for each pH values (5.5, 6.5, 7.4) while changing one of the following conditions:

i) The reaction was performed at 45°C.
ii) 2-(Boc-amino) ethanethiol (28 µL, 161.0 µmol, 10 equiv.) was used.
iii) **1Tz** (0.5 mg, 1.6 µmol) was used.
iv) $N_2$ bubbling was applied while running the reaction.

**Table S2.** Effect of temperature, thiol concentration, overall concentration, and $N_2$ bubbling on the **TeTEx** conversion after 1 hour reaction time. Each column reflects the condition varied from the standard reaction conditions.

| pH [a] | Standard Conditions [b] | 45°C | 10 equiv. [SH] | 1.6 mM Conc. | $N_2$ Bubbling |
|---|---|---|---|---|---|
| 5.5 | 66% | 81% | 68% | 33% | 61% |
| 6.5 | 75% | 74% | 81% | 63% | 93% |
| 7.4 | 72% | 61% | 75% | 83% | 92% |

[a] Buffers used are shown in **Table S1**. [b] The standard conditions are indicated above.

**Table S3.** Effect of solvent on the **TeTEx** between **1Tz** and 2-(Boc-amino) ethanethiol (1.0 equiv.) at 21°C, while bubbling with $N_2$ after 30 min reaction time.

| Solvent | Conversion |
|---|---|
| MeOH | 4% |
| MeOH with NaCl [a] | 40% |
| MilliQ/MeOH [b] | >2% |
| PBS/MeOH [c] | 42% |
| ACN | 50% |
| MilliQ/ACN [b] | 77% |
| PBS/ACN [c] | 98% |
| DMSO | 12% |
| MilliQ/DMSO [b] | 7% |
| PBS/DMSO [c] | 92% |
| THF | 4% |
| THF [d] | 2% |
| MilliQ/THF [b] | 2% |
| THF with LiBr [a] | >2% |
| PBS/THF [c] | 84% |

[a] Saturated organic solvent mixtures with salt. [b] MilliQ/organic solvent solutions (1:1 v/v). [c] PBS (pH 6.5)/organic solvent mixtures (1:2 v/v). [d] No $N_2$ bubbling.

DOI: 10.1039/x0xx00000x

## 5.4. Postulated reaction mechanism of TeTEx with 3-thiomethyl tetrazines

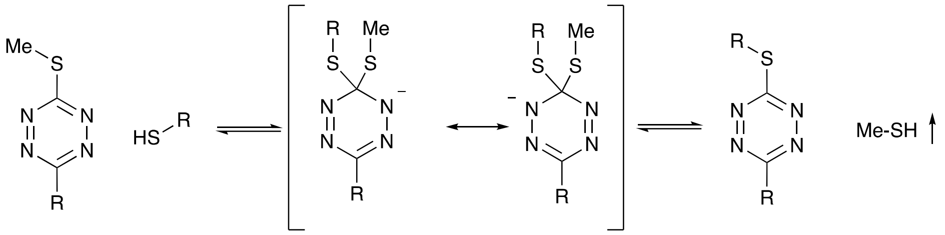


**Scheme S1.** Postulated mechanisms of **TeTEx**. The thiol nucleophile attacks the thiomethyl bearing carbon and the negative charge is stabilized by resonance, followed by removal of the leaving group, thiomethyl.

## 5.5. Representative selection of HPLC traces during the optimization of TeTEx conditions

### *5.5.1. HPLC trace of **TeTEx** at the determined optimal conditions with excess thiol.*

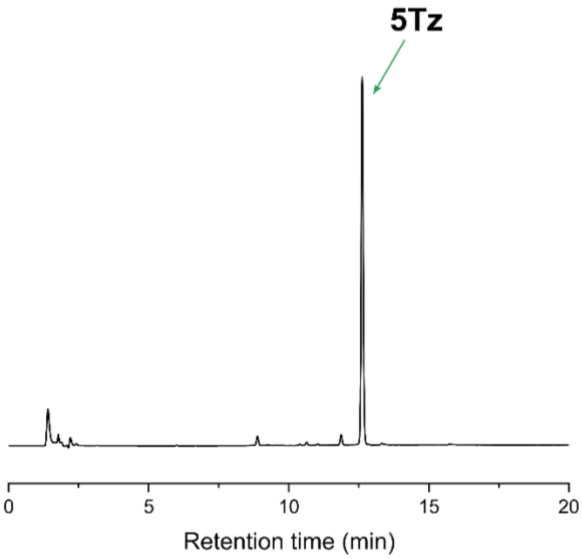


**Figure S8.** HPLC trace **TeTEx** between **1Tz** and 2-(Boc-amino) ethanethiol (10.0 equiv.) at pH 6.5, 21°C, while bubbling with $N_2$ after 1 h reaction time.

### *5.5.2. HPLC trace of **TeTEx** at the determined optimal conditions with stoichiometric amounts of thiol.*

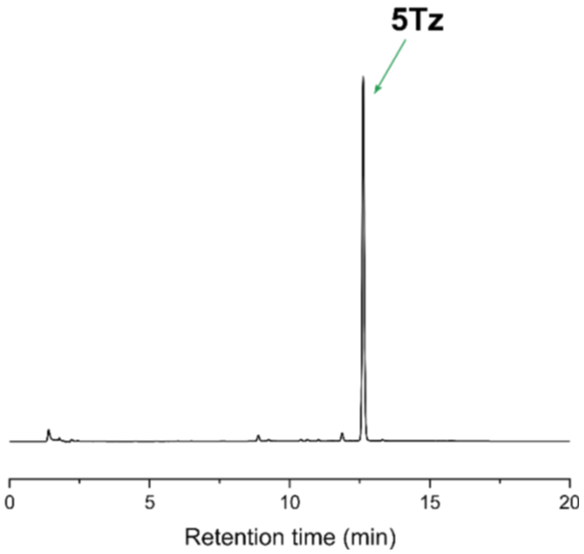


**Figure S9.** HPLC trace of **TeTEx** between **1Tz** and 2-(Boc-amino) ethanethiol (1.0 equiv.) at pH 6.5, 21°C, while bubbling with $N_2$ after 1 h reaction time.

### *5.5.3. HPLC trace of **TeTEx** at the determined optimal conditions at 200 µM concentration.*

After the optimization, **TeTEx** was performed with 1Tz (200 µM) and 2-(Boc-amino) ethanethiol (200 µM) using the optimal conditions.

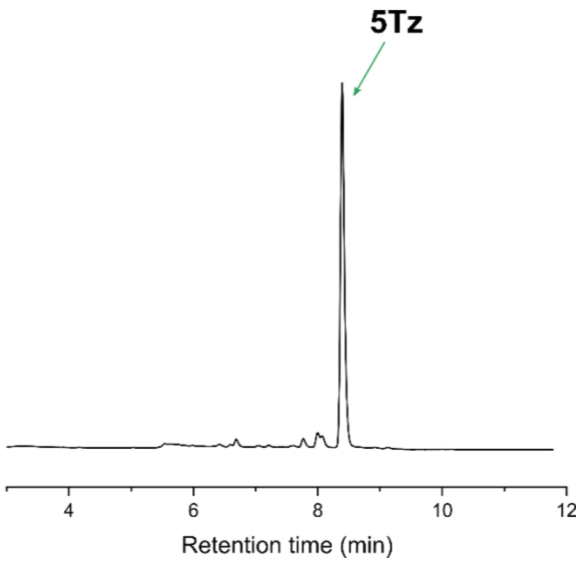


**Figure S10.** HPLC trace of **TeTEx** between **1Tz** (200 µM) and 2-(Boc-amino) ethanethiol (1.0 equiv.) in PBS (pH 6.5)/DMSO (1:1 v/v), 21°C, while bubbling with $N_2$ after 30 min reaction time. The trace was taken with a shortened HPLC method.

DOI: 10.1039/x0xx00000x

### *5.5.4. Tetrazine decomposition at pH 7.4 when excess thiol or increased temperature was employed.*

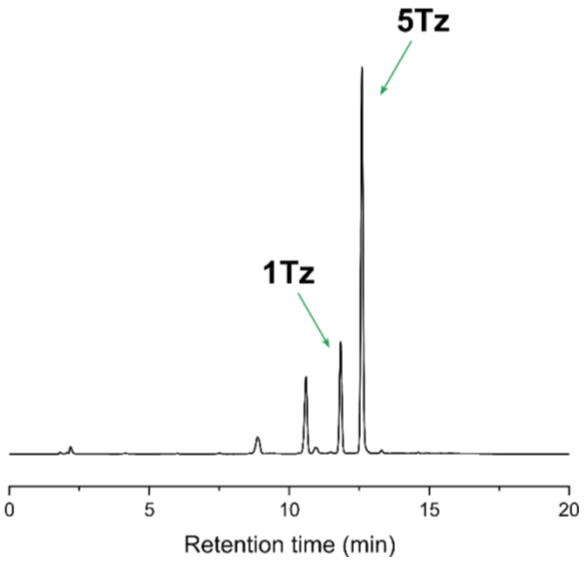


**Figure S11.** HPLC trace **TeTEx** between **1Tz** and 2-(Boc-amino) ethanethiol (10.0 equiv.) at pH 7.4 after 2 h reaction time. The appearance of new peaks below tr = 11.5 indicate decomposition of the tetrazine.

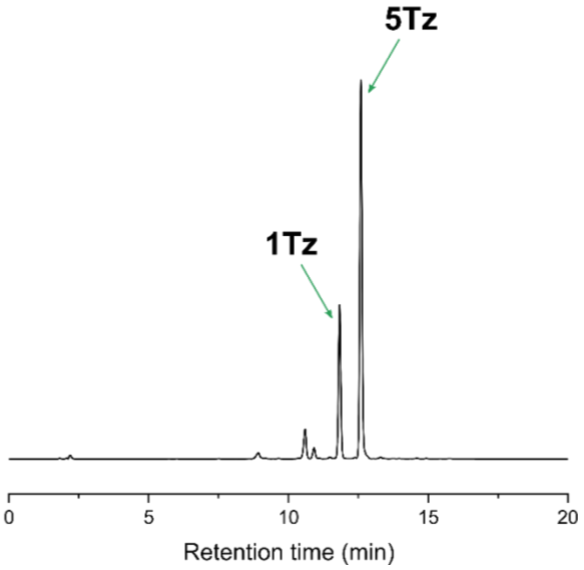


**Figure S12.** HPLC trace **TeTEx** between **1Tz** and 2-(Boc-amino) ethanethiol (5.0 equiv.) at pH 7.4 after 2 h reaction time at 45°C. The appearance of new peaks below tr = 11.5 indicate decomposition of the tetrazine.

## 6. Scope determination for TeTEx

### 6.1. Compatibility of 1Tz with TCEP

**1Tz** (0.5 mg, 1.6 µmol) was dissolved in 1 mL PBS (pH 6.5)/ACN solution (1.6 mM, 1:1 v/v). Then 10 µL of a 160 mM solution of TCEP were added and the stability of the compound was monitored by LC-MS for 24 h at 21°C. The mixture was allowed to sit overnight open to air.

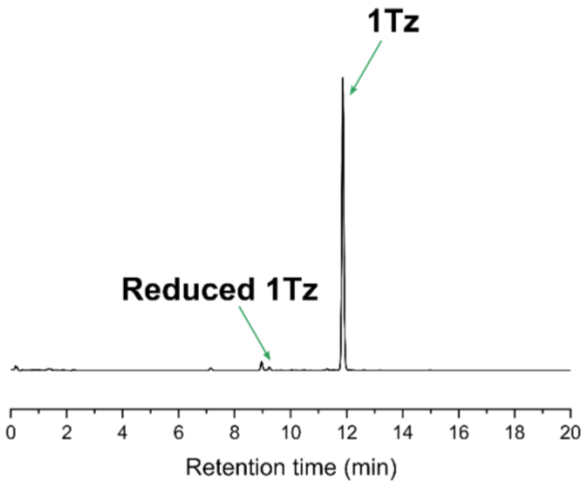


**Figure S13.** HPLC trace of **1Tz** and TCEP after 24 h. An LCMS trace was taken of the mixture and it was found that the large peak (tr = 6.98 min) corresponds to the **1Tz** (MS (ESI): calculated for $[C_{13}H_{22}N_5O_2S]^+$: m/z 311.4, found: m/z 297.0 (removal of $N_2$ via unknown mechanism), while the dihydrotetrazine was not ionizable on LC-MS.

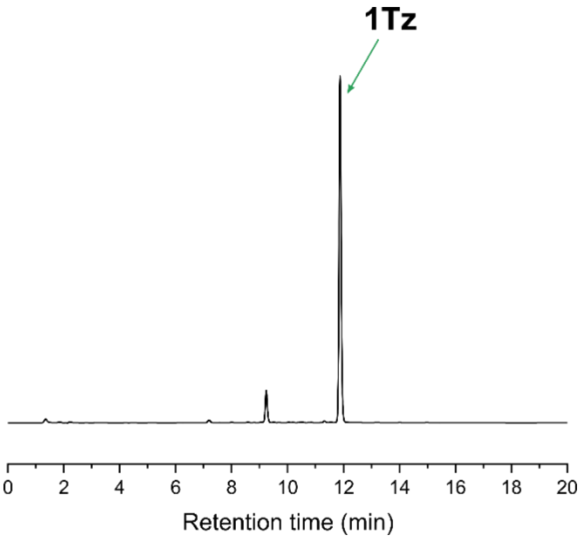


**Figure S14.** HPLC trace of mixture between **1Tz** and TCEP after 24 h open to air.

## 6.2. Dependance of thiol nucleophilicity in the efficiency of TeTEx

**1Tz** (0.5 mg, 1.6 µmol) was dissolved in a 1 mL citric acid buffer pH 5.5/ACN solution (1.6 mM, 1:1 v/v). Thiol (1.0 equiv.) and TCEP (0.9 mg, 3.2 µmol, 2.0 equiv.) were added and the mixture was incubated at 21°C and 300 rpm while bubbling with $N_2$. The reaction was analyzed via LC-MS after 2 h.

DOI: 10.1039/x0xx00000x

### *6.2.1. Glutathione*

The protocol above was performed using glutathione (0.5 mg, 1.6 µmol, 1.0 equiv.). The mixture was allowed to sit overnight open to air.

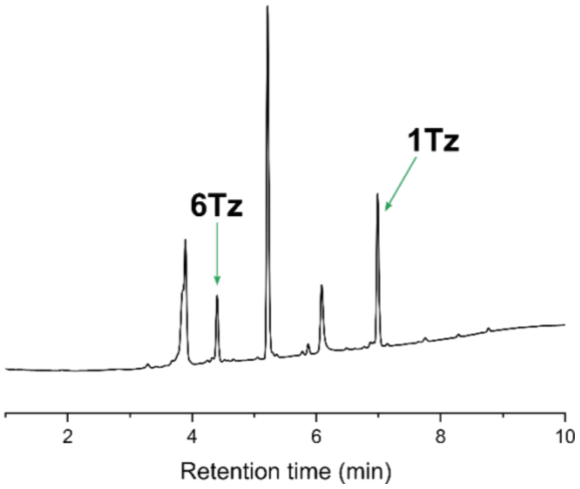


**Figure S15.** LC-MS trace of **TeTEx** between **1Tz** and glutathione after 2 h. From left to right, the first peak (tr = 3.89 min) is the reduced **6Tz** (MS (ESI): calculated for $[C_{22}H_{37}N_8O_8S]^+$: m/z 573.2, found: m/z 573.2), the second peak (tr = 4.40 min) is the **6Tz** (MS (ESI): calculated for $[C_{22}H_{35}N_8O_8S]^+$: m/z 571.2, found: m/z 571.2) and the last peak (tr = 6.99 min) is **1Tz** as determined from previous LCMS analysis. The remaining two peaks were not identifiable by MS but are hypothesized to be the glutathione and reduced **1Tz**.

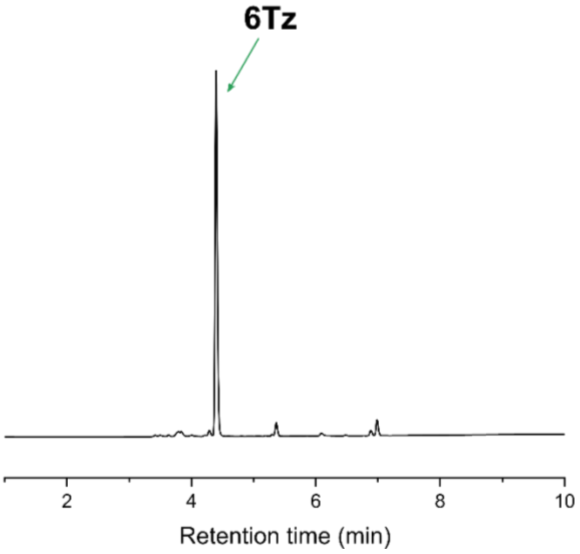


**Figure S16.** LC-MS trace of **TeTEx** between **1Tz** and glutathione after 24 h open to air.

 DOI: 10.1039/x0xx00000x

### 6.2.2. tert-Butylthiol

The protocol above was performed using *tert*-butylthiol (0.2 µL, 1.6 µmol, 1.0 equiv.).

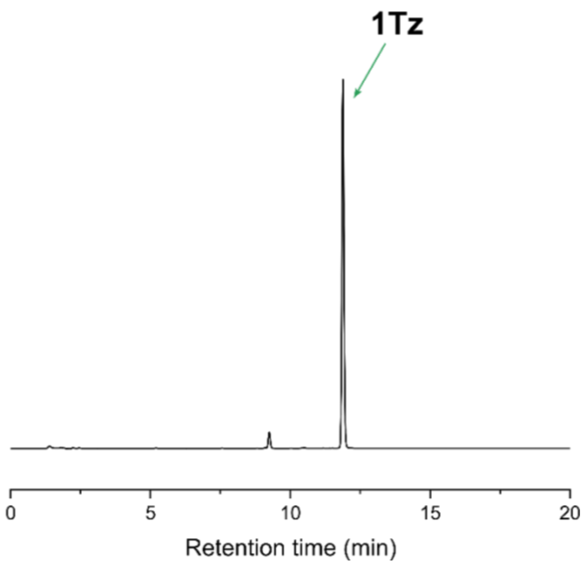


**Figure S17.** HPLC trace of **TeTEx** between **1Tz** and *tert*-butyl thiol. No product was observed due to steric hindrance.

### 6.2.3. Thioacetic acid

The protocol above was performed using thioacetic acid (0.1 µL, 1.6 µmol, 1.0 equiv.).

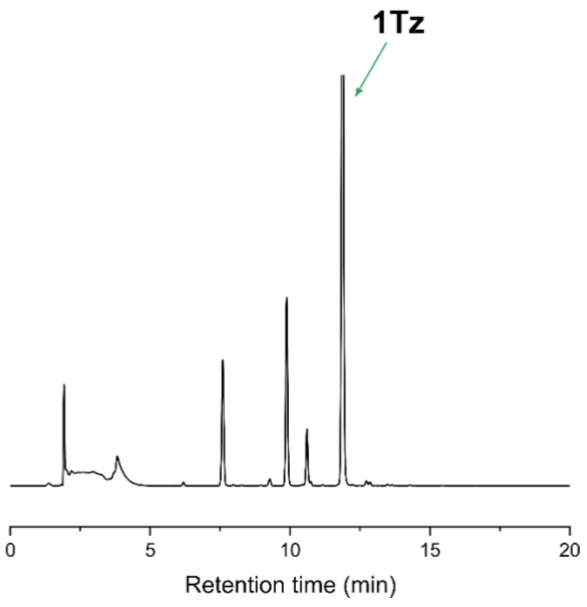


**Figure S18.** HPLC trace of **TeTEx** between **1Tz** and thioacetic acid. No product is observed due to decomposition.

DOI: 10.1039/x0xx00000x

### *6.2.4. Thiophenol*

The protocol above was performed using thiophenol (0.2 µL, 1.6 µmol, 1.0 equiv.).

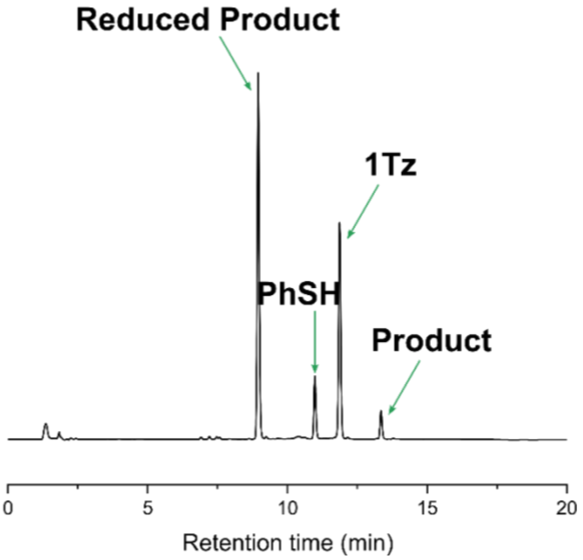


**Figure S19.** HPLC trace of **TeTEx** between **1Tz** and thiophenol. After the product is formed it undergoes reduction and eventual decomposition. From left to right, the first peak (tr = 8.96 min) is the reduced product (MS (ESI): calculated for $[C_{18}H_{26}N_5O_2S]^+$: m/z 376.2, found: m/z 376.1), the second peak (tr = 10.98 min) is the thiophenol as determined by previous HPLC analysis of the starting compound, the third peak (tr = 11.87 min) is **1Tz** as determined from previous HPLC analysis of the pure compound, and the final peak (tr = 13.35 min) is the product (MS (ESI): calculated for $[C_{18}H_{24}N_5O_2S]^+$: m/z 374.2, found: m/z 359.0 (removal of $N_2$ via unknown mechanism)).

## 6.3. Tetrazine electronic properties effect on TeTEx efficiency and selectivity

125 µL of a 2 mM solution of peptide **AKWSGCL** in PBS (pH 6.5) was mixed with 125 µL of a 1 mM solution of TCEP in MilliQ and was allowed to mix for 10 min at 350 rpm. 250 µL of a 1 mM solution of functional **1Tz – 4Tz** in ACN was added in the mixture and the solution was mixed at 37°C and 350 rpm for 1 hour. The reaction was analyzed via MALDI-TOF.

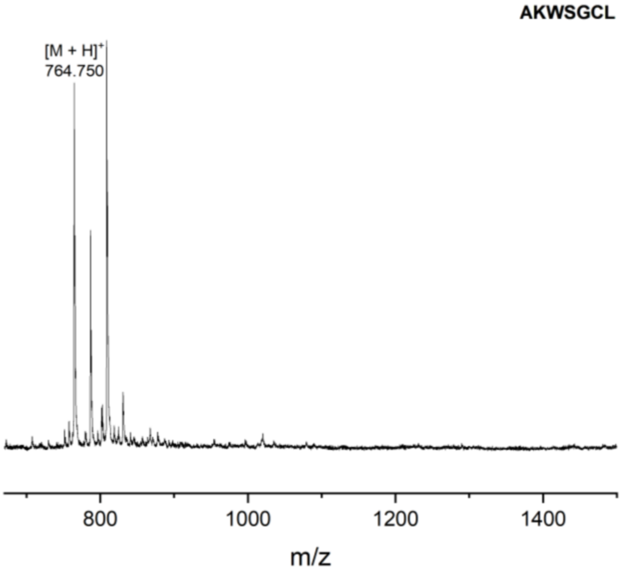


**Figure S20.** MALDI-TOF trace of peptide **AKWSGCL**. Proton and sodium adducts are visible.

DOI: 10.1039/x0xx00000x

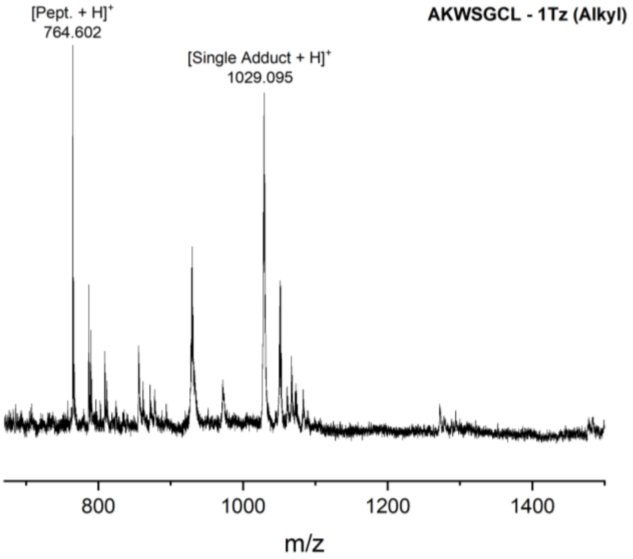


**Figure S21.** MALDI-TOF trace of **TeTEx** between **1Tz** and peptide **AKWSGCL**. Proton and sodium adducts of the free peptide are still visible. Single protonated and sodiated **1Tz**-peptide adducts are visible.

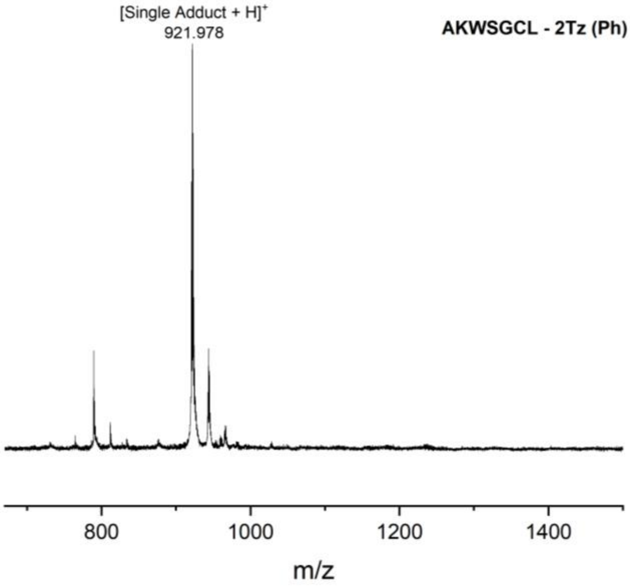


**Figure S22.** MALDI-TOF trace of **TeTEx** between **2Tz** and peptide **AKWSGCL**. The sodium adduct of the free peptide is still visible. Single protonated and sodiated **2Tz**-peptide adducts are visible.

DOI: 10.1039/x0xx00000x

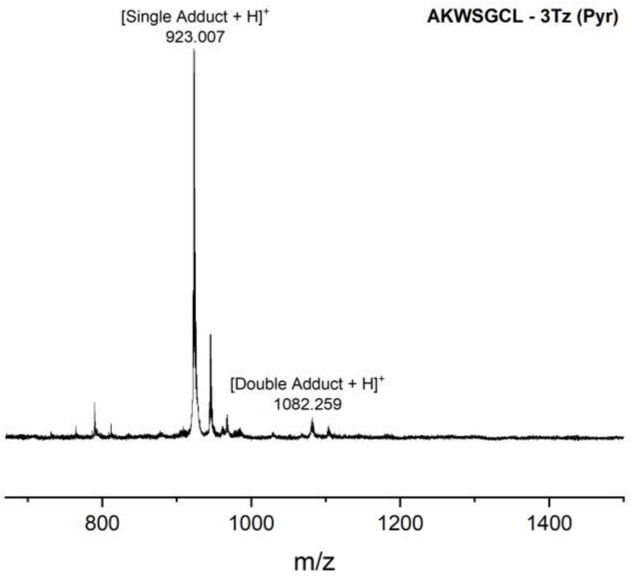


**Figure S23.** MALDI-TOF trace of **TeTEx** between **3Tz** and peptide **AKWSGCL**. Single protonated and sodiated **3Tz**-peptide adducts are visible. A small amount of double **3Tz**-peptide adduct is also present.

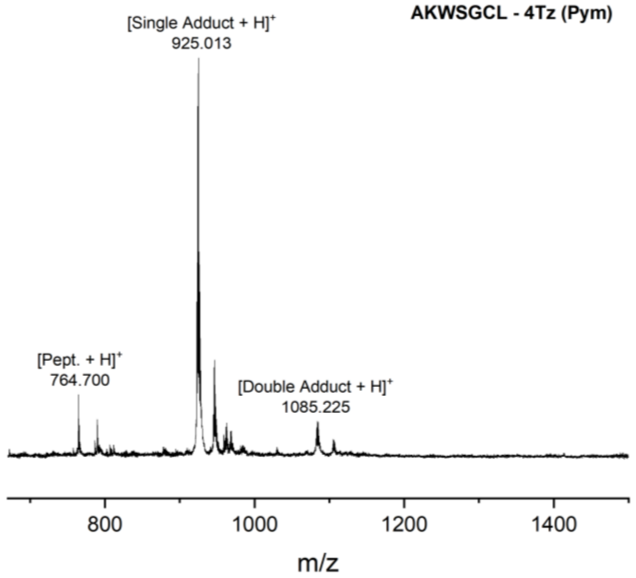


**Figure S24.** MALDI-TOF trace of **TeTEx** between **4Tz** and peptide **AKWSGCL**. Proton and sodium adducts of the free peptide are still visible. Single protonated and sodiated **4Tz**-peptide adducts are visible. The double **3Tz**-peptide adduct is also present.

## 7. Investigation of trans-click reaction

**5Tz** (0.7 mg, 1.6 µmol) was dissolved in a 1 mL PBS (pH 6.5)/ACN solution (1.6 mM, 1:1 v/v). Glutathione (0.5 mg, 1.6 µmol, 1.0 equiv.) and TCEP (0.5 mg, 1.6 µmol, 1.0 equiv.) were added and the mixture was incubated at 21°C and 300 rpm. The reaction was analyzed via HPLC after 2 and 24 h.

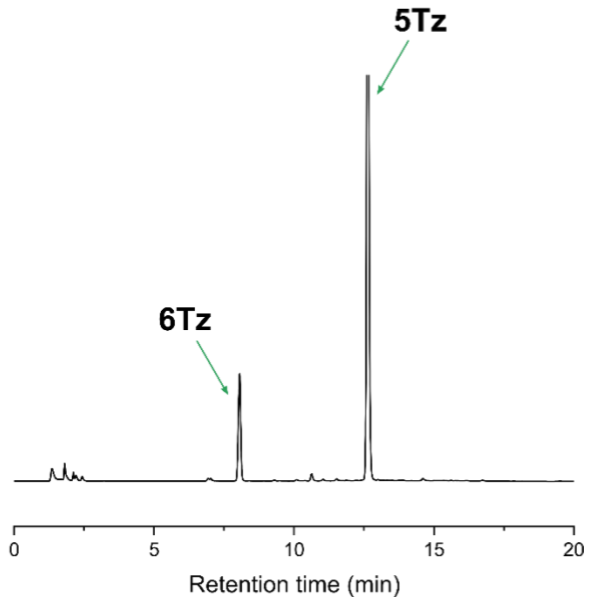


**Figure S25.** HPLC trace of trans-click reaction between **5Tz** and glutathione (1.0 equiv.) after 2 h reaction time.

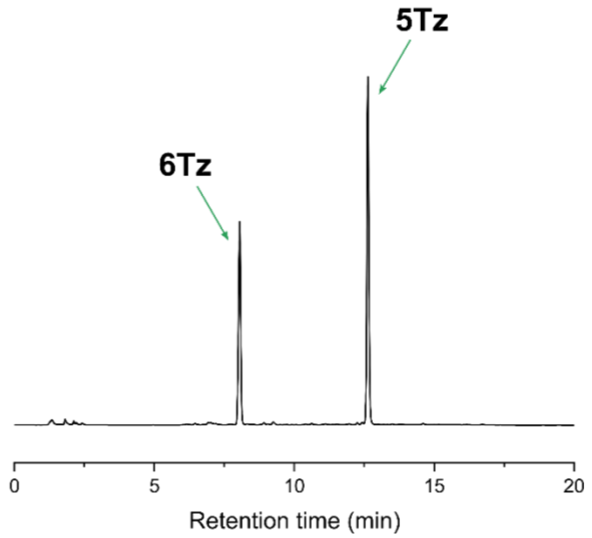


**Figure S26.** HPLC trace of trans-click reaction between **5Tz** and glutathione (1.0 equiv.) after 24 h reaction time.

## 8. Fluorescent assay for kinetic determination

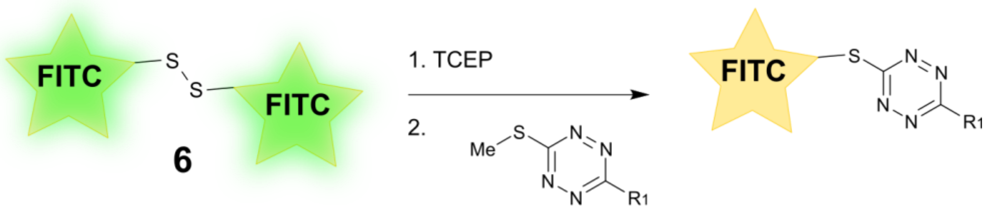


**Scheme S2.** Schematic representation of kinetic assay monitoring the quenching of FITC fluorescence *via* **TeTEx** with tetrazines **1Tz – 4Tz**.

50 µL of a solution of **6** (aiming for 5 µM, later the concentration was determined to be between 6-7 µM with a fluorescence calibration line) in PBS (pH 6.5), 20 µL of PBS (pH 6.5) and 10 µL of a 250 µM solution of TCEP in MilliQ were mixed. After 10 min, 20 µL solution of tetrazines **1Tz – 4Tz** (1 equiv) in ACN was added and the fluorescence intensity ($\lambda_{em}$ = 519 nm) was

monitored every 2 min for 46 min. The experiment was performed in triplicate. A calibration line (**Figure S1**) (FITC-cystamine concentrations: 0, 1.25, 2.5, 3.75, 5 µM) was used to calculate the average unreacted FITC concentration from the three replicates. Plotting the 1/concentration against time allowed us to determine the second-order reaction rate ($k_2$) as the slope of the line.

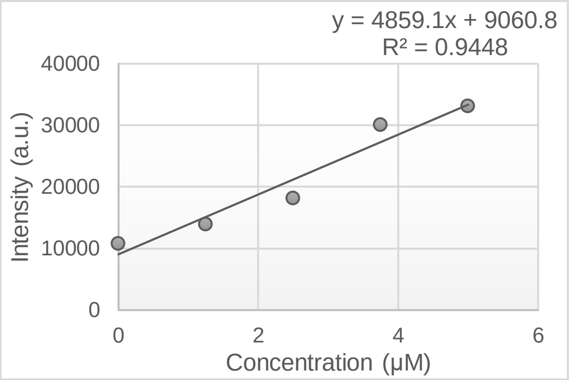


**Figure S27.** Calibration line constructed by measuring the fluorescence intensity of compound **6** at 0, 1.25, 2.5, 3.75, 5 µM.

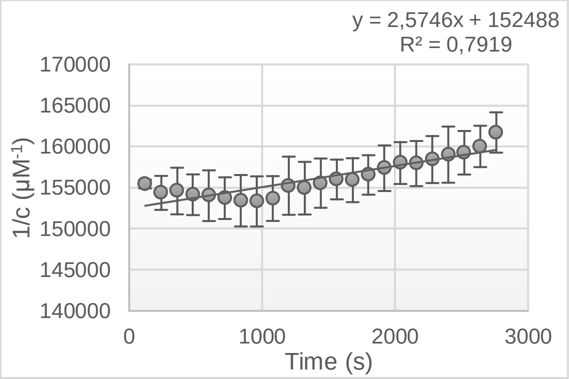


**Figure S28.** 1/concentration plotted against time for **TeTEx** between **6** and **1Tz**. Each measurement was done in triplicate.

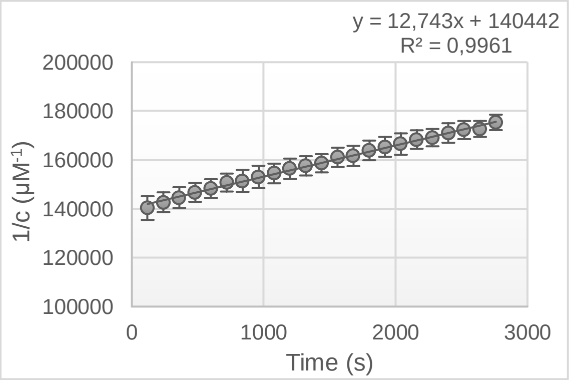


**Figure S29.** 1/concentration plotted against time for **TeTEx** between **6** and **2Tz**. Each measurement was done in triplicate.

 DOI: 10.1039/x0xx00000x

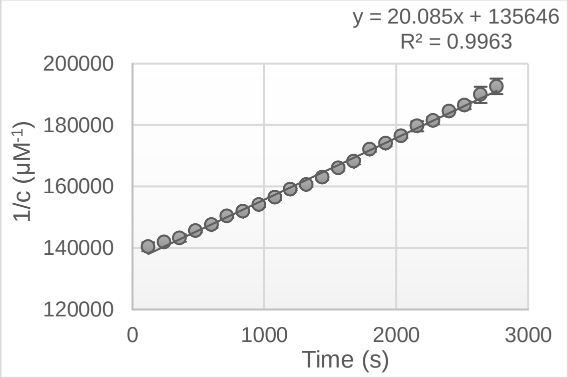


**Figure S30.** 1/concentration plotted against time for **TeTEx** between **6** and **3Tz**. Each measurement was done in triplicate.

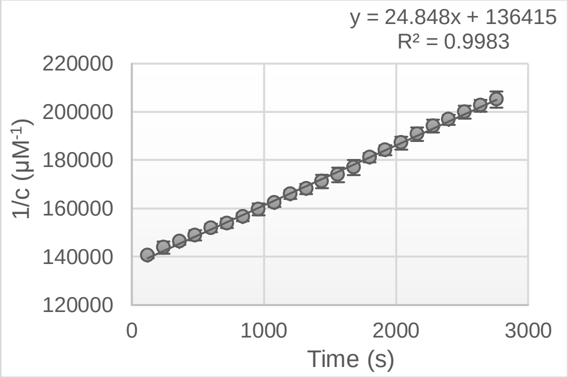


**Figure S31.** 1/concentration plotted against time for **TeTEx** between **6** and **4Tz**. Each measurement was done in triplicate.

## 9. Cysteine electrophiles

**Table S4.** Cysteine electrophiles and their reaction rates, found in literature.[2–8]

| substrate | reaction rate / $M^{-1}$ $s^{-1}$ | reference |
|---|---|---|
| 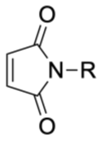 | 10 - 1000 | ACS Chem Biol., 2015, 10, 1026-1033 |
| 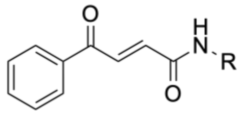 | ~11 | Nat. Comm. 2016, 7, 13128 |
| 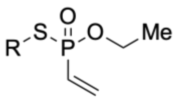 | 0.0021 - 0.074 | J. Am. Chem. Soc. 2020, 142, 9544-9552 |
| 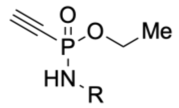 | 0.62 | Angew. Chem. Int. Ed., 2019, 58,11625-11630 |
| 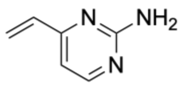 | 375 | **Chem. Sci.**, 2021,**12**, 9060-9068 |
| 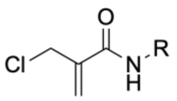 | 1.17 | **Chem. Sci.**, 2021,**12**, 13321-13330 |
| 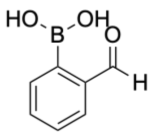 | 238 | **Chem. Sci.**, 2016,**7** ,5052-5058 |

## 10. Investigation of Click'n Lock

100 µL of a 5mM solution of **1Tz – 4Tz** in ACN, 385 µL ACN and 500 µL PBS (pH 6.5) were mixed together. 15 µL of a 100 mM solution of dienophile in ACN was added and the mixture was stirred at 37°C and 300 rpm for 24 h. The reaction was monitored via LC-MS.

## 10.1. Hydrolysis of 1,2-dihydropyridazine upon IEDDA

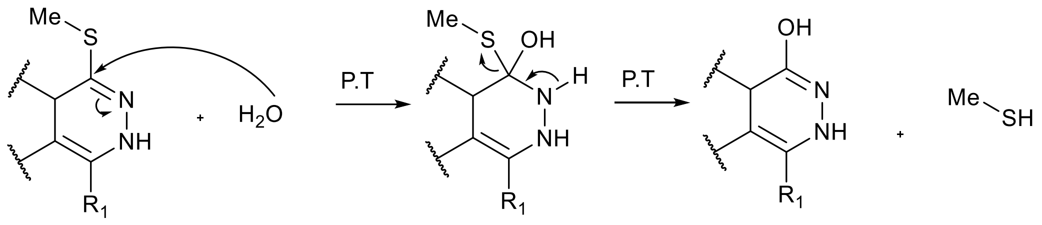


**Scheme S3.** Postulated mechanisms displaying the hydrolysis of the 1,2-dihydropyridazine upon IEDDA reaction of **2Tz – 3Tz** with *exo*-5-norbornenecarboxylic acid (P.T = proton transfer).

## 10.2. Representative selection of HPLC traces displaying lock via IEDDA

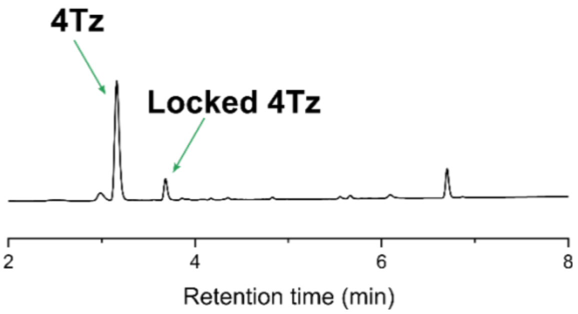


**Figure S32.** LC-MS trace showing IEDDA reaction between **4Tz** and PVE (10.0 equiv.). Little conversion is observed.

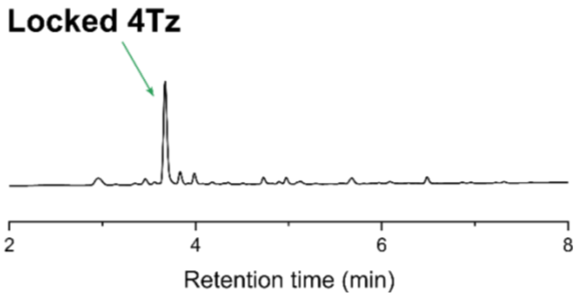


**Figure S33.** LC-MS trace shows that IEDDA reaction between **4Tz** and norbornadiene led to quantitative lock.

 DOI: 10.1039/x0xx00000x

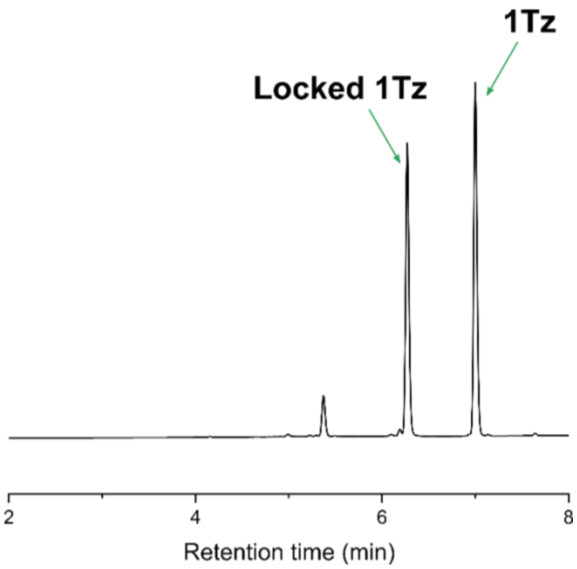


**Figure S34.** LC-MS trace shows that IEDDA reaction between **1Tz** and BCN led to lock.

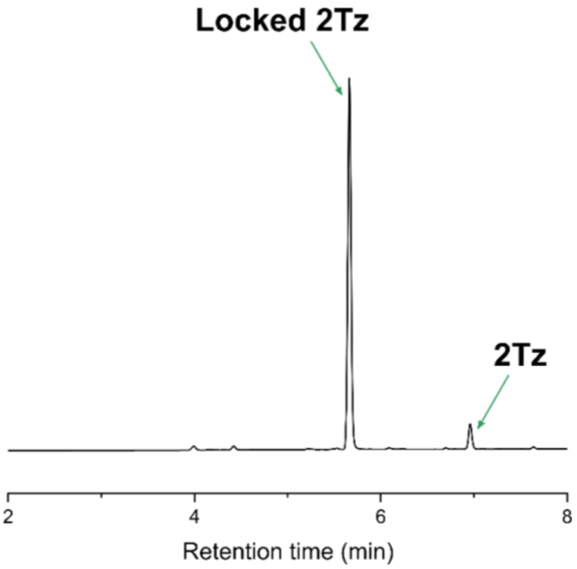


**Figure S35.** LC-MS trace shows that IEDDA reaction between **2Tz** and BCN led to completed lock (> 90%).

DOI: 10.1039/x0xx00000x

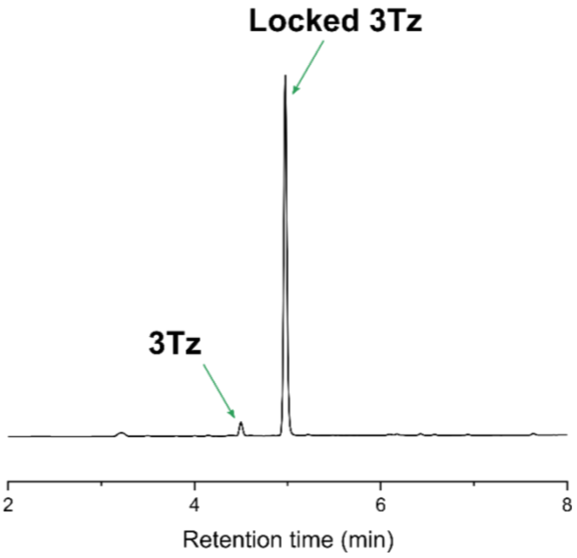


**Figure S36** LC-MS trace shows that IEDDA reaction between **3Tz** and BCN led to completed lock (> 90%).

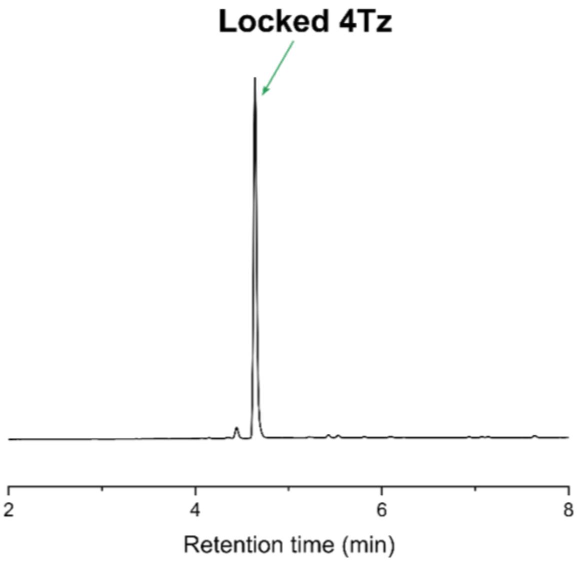


**Figure S37.** LC-MS trace shows that IEDDA reaction between **4Tz** and BCN led to quantitative lock (> 90%).

### 10.3. Click'n Lock demonstrated on peptide GFRDGCA

250 µL of a 2 mM solution of peptide **GFRDGCA** in PBS (pH 6.5) and 250 µL of a 1 mM solution of TCEP in MilliQ were allowed to stir for 10 min at 350 rpm. 500 µL of a 1 mM solution of **1Tz** in ACN was added and the solution was mixed at 37°C and 350 rpm overnight. The reaction was monitored via MALDI-TOF. Once **TeTEx** was completed, 180 µL of modified peptide was mixed with 5 µL of a 100 mM solution of DTT in ACN or 100 µL of a 5 mM solution of BCN in PBS (pH 6.5) and the reactions were allowed to proceed at 350 rpm. Once the lock was complete, according to MALDI-TOF, 5 µL of a 100 mM solution of DTT in ACN was added to the second sample and the reaction was allowed to stir at 350 rpm overnight. The two reactions were analyzed by MALDI-TOF. For reproducibility, the experiment was performed again as described with a peptide concentration of 360 µM.

 DOI: 10.1039/x0xx00000x

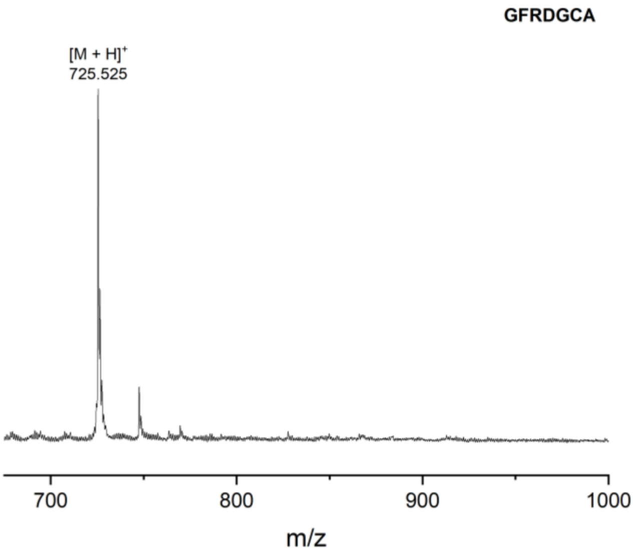


**Figure S38.** MALDI-TOF trace of peptide **GFRDGCA**. Proton and sodium adduct of the peptide are visible.

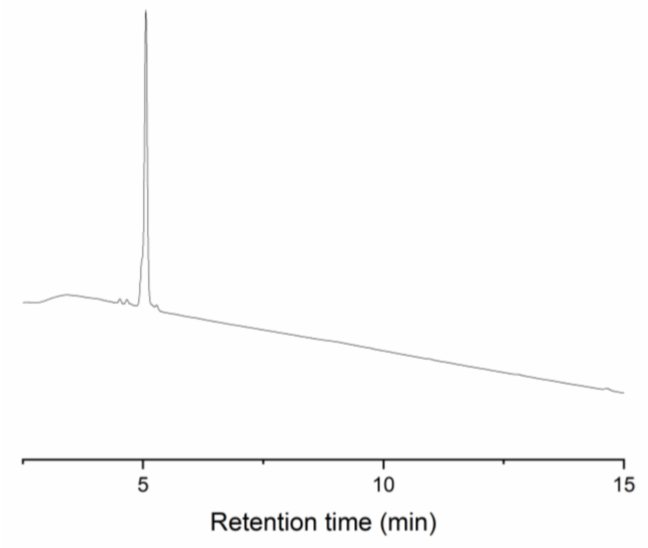


**Figure S39.** HPLC trace of peptide **GFRDGCA**.

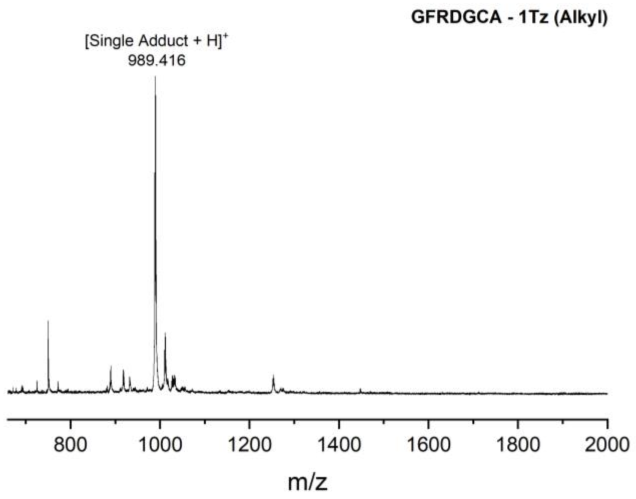


**Figure S40.** MALDI-TOF trace of **TeTEx** between **1Tz** and peptide **AKWSGCL**. The sodium adduct of the free peptide is still visible. Single protonated and sodiated **1Tz**-peptide adducts are visible.

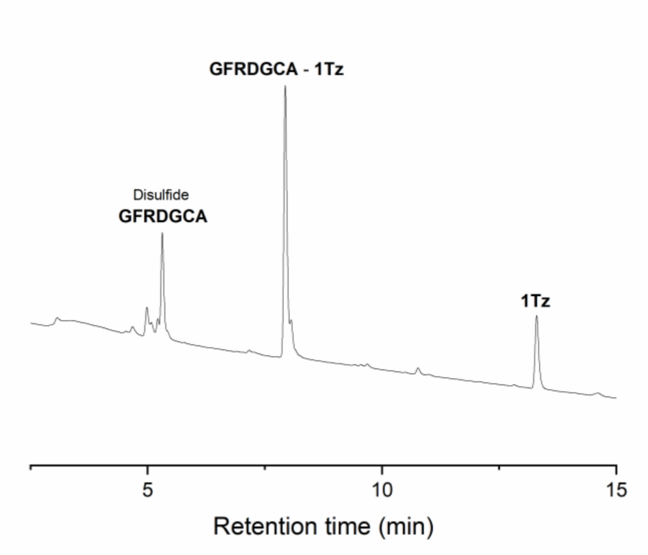


**Figure S41.** HPLC trace of peptide **GFRDGCA-1Tz** adduct. Some free **1Tz** and disulfide form of peptide is present. The disulfide is unreactive towards **TeTEx**.

 DOI: 10.1039/x0xx00000x

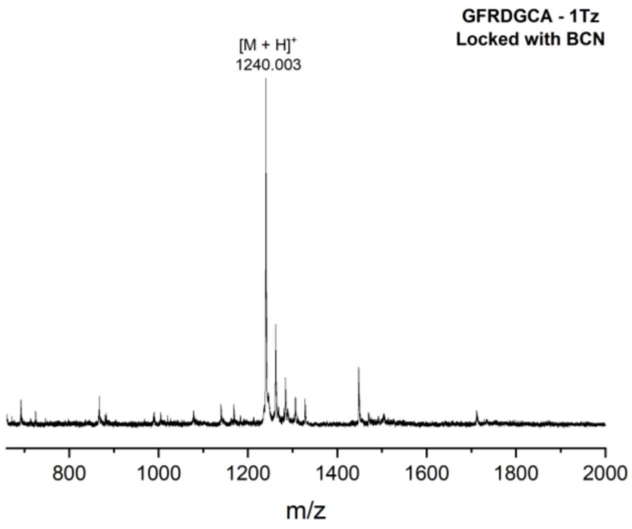


**Figure S42.** MALDI-TOF trace of IEDDA cycloaddition between **AKWSGCL - 1T** and BCN. Complete lock is observed.

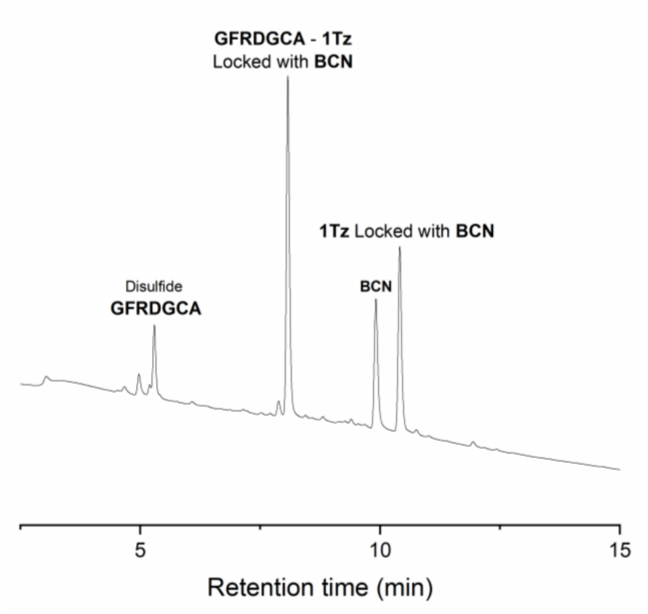


**Figure S43.** HPLC trace of peptide **GFRDGCA-1Tz** adduct locked with BCN. The free **1Tz** has also reacted with BCN.

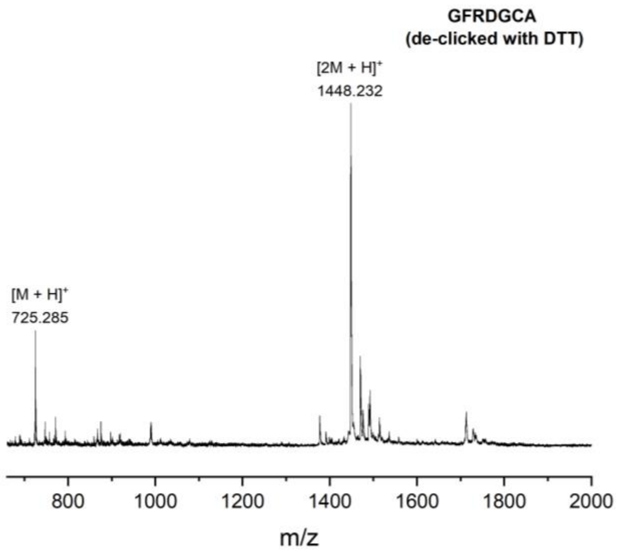


**Figure S44.** MALDI-TOF trace of **AKWSGCL** - **1T** after de-click reaction with DTT. Complete de-click reaction is observed.

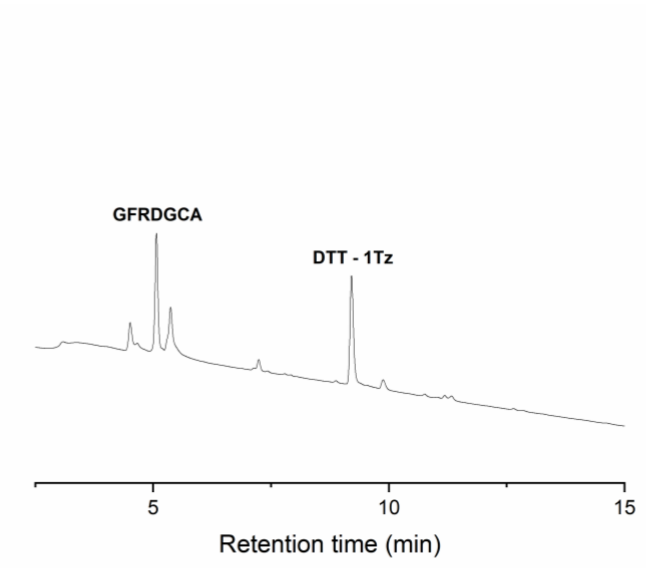


**Figure S45.** HPLC trace of peptide **GFRDGCA** after de-click reaction with DTT. The **1Tz**-DTT adduct is also visible.

DOI: 10.1039/x0xx00000x

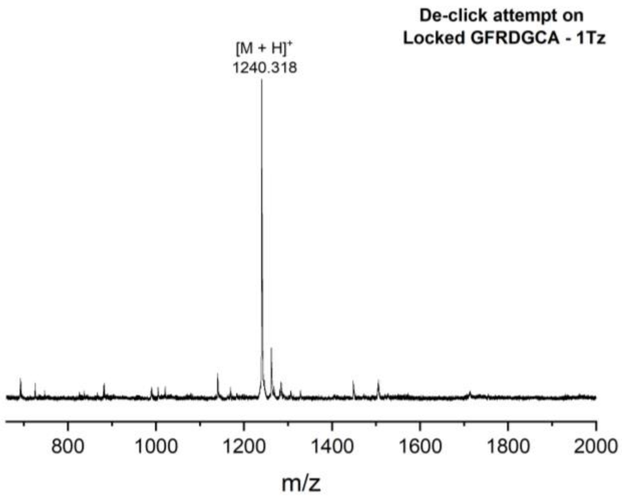


**Figure S46.** MALDI-TOF trace of locked **AKWSGCL** - **1T** after de-click reaction with DTT. No de-click reaction is observed.

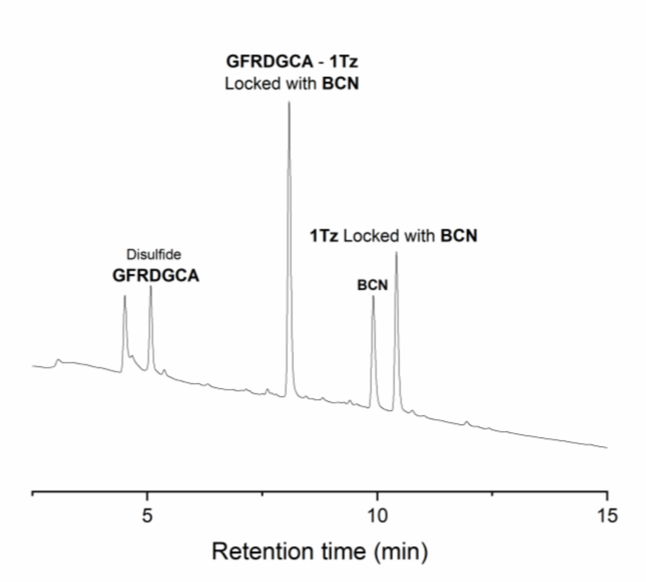


**Figure S47.** HPLC trace of peptide **GFRDGCA-1Tz** locked with BCN after de-click reaction with DTT. No de-click reaction is observed.

## 10.4. Click'n Lock demonstrated with small molecule thiol

**1Tz** (3 mg, 10µmol, 1 equiv.) was dissolved in PBS (pH 6.5)/ACN mixture (1:1 v/v) and 2-(Boc-amino) ethanethiol (8.7 µL, 10 µmol, 1 equiv.) was added. The solution was allowed to stir for 1 hour while bubbling with $N_2$ at 350 rpm. The reaction was monitored via LC-MS. Once **TeTEx** was completed, 100 µL of a 200 mM solution of BCN in PBS (pH 6.5) was added and the reaction was allowed to proceed at 37°C overnight at 350 rpm. The solution was left at 37°C for 4 days at 350 rpm, and the stability of the compound was monitored via LC-MS. Then 100 µL of a 500 mM solution of glutathione in PBS (pH 6.5) was added to the sample and the reaction was allowed to stir at 37°C for 3 days at 350 rpm, and the stability of the compound was monitored via LC-MS.

 DOI: 10.1039/x0xx00000x

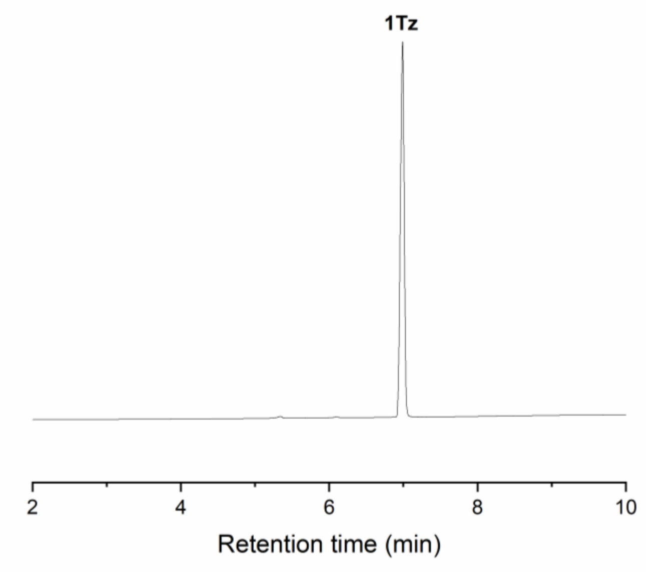


**Figure S48.** HPLC trace of **1Tz.**

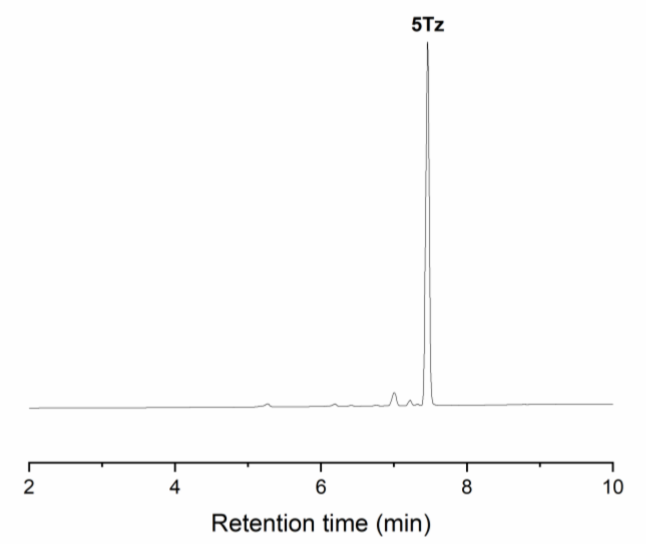


**Figure S49.** HPLC trace of **5Tz** after **TeTEx** between 1Tz and 2-(Boc-amino) ethanethiol**.**

DOI: 10.1039/x0xx00000x

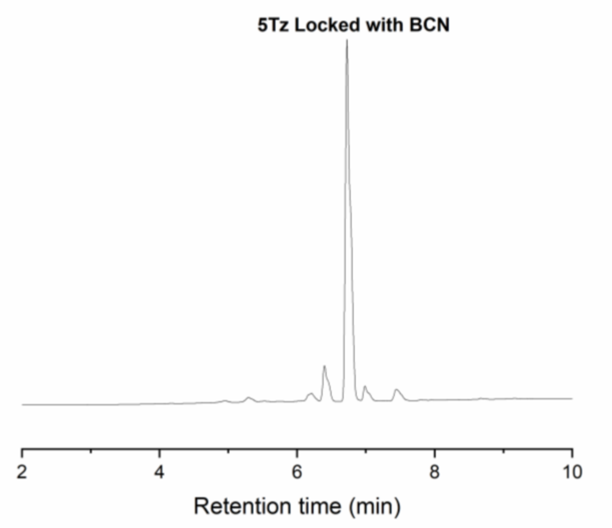


**Figure S50.** HPLC trace of **5Tz** after lock with BCN.

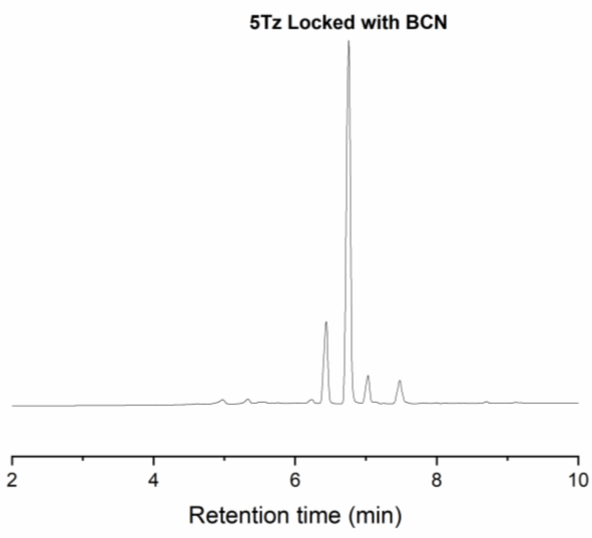


**Figure S51.** HPLC trace of **5Tz** after lock with BCN and incubated at 37°C for 4 days.

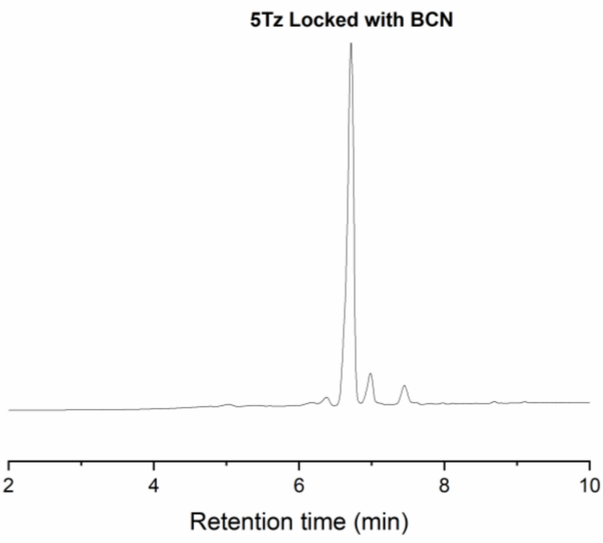


**Figure S52.** HPLC trace of **5Tz** after lock with BCN and incubated at 37°C for 3 days in the presence of glutathione.

 DOI: 10.1039/x0xx00000x

## 11. NMR Spectra

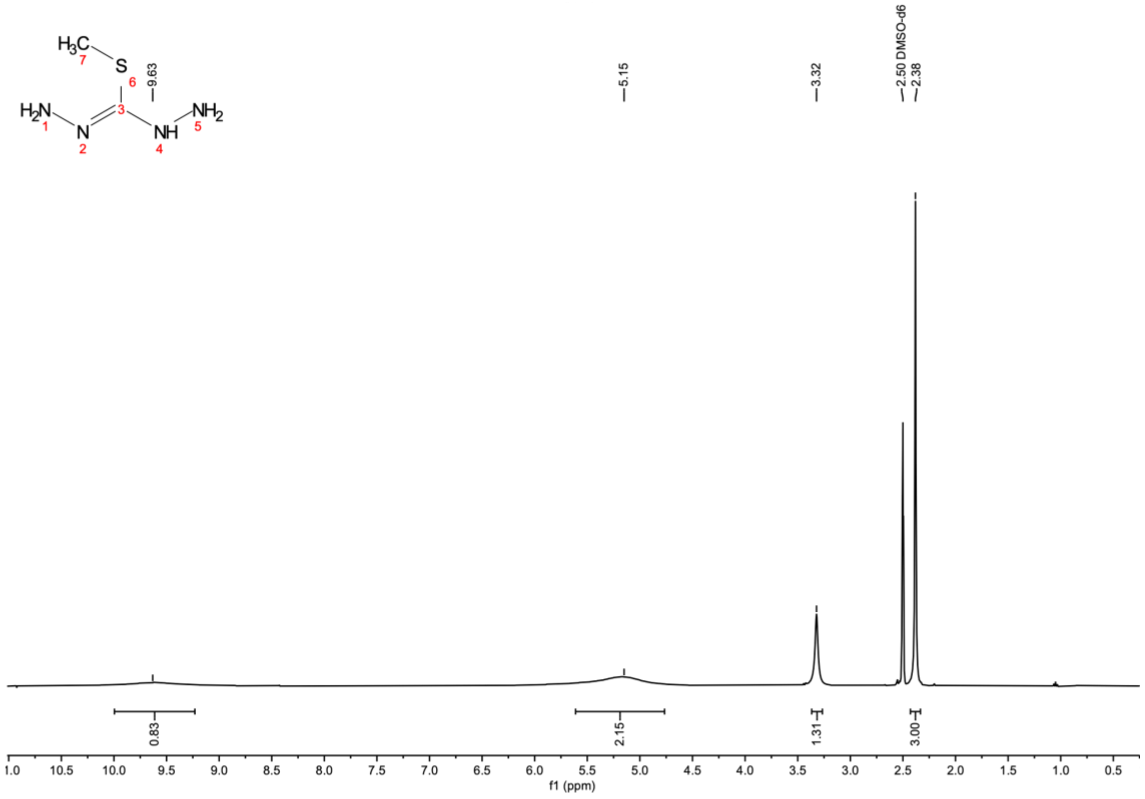

**Figure S53.** $^{1}$H-NMR of compound **1**.

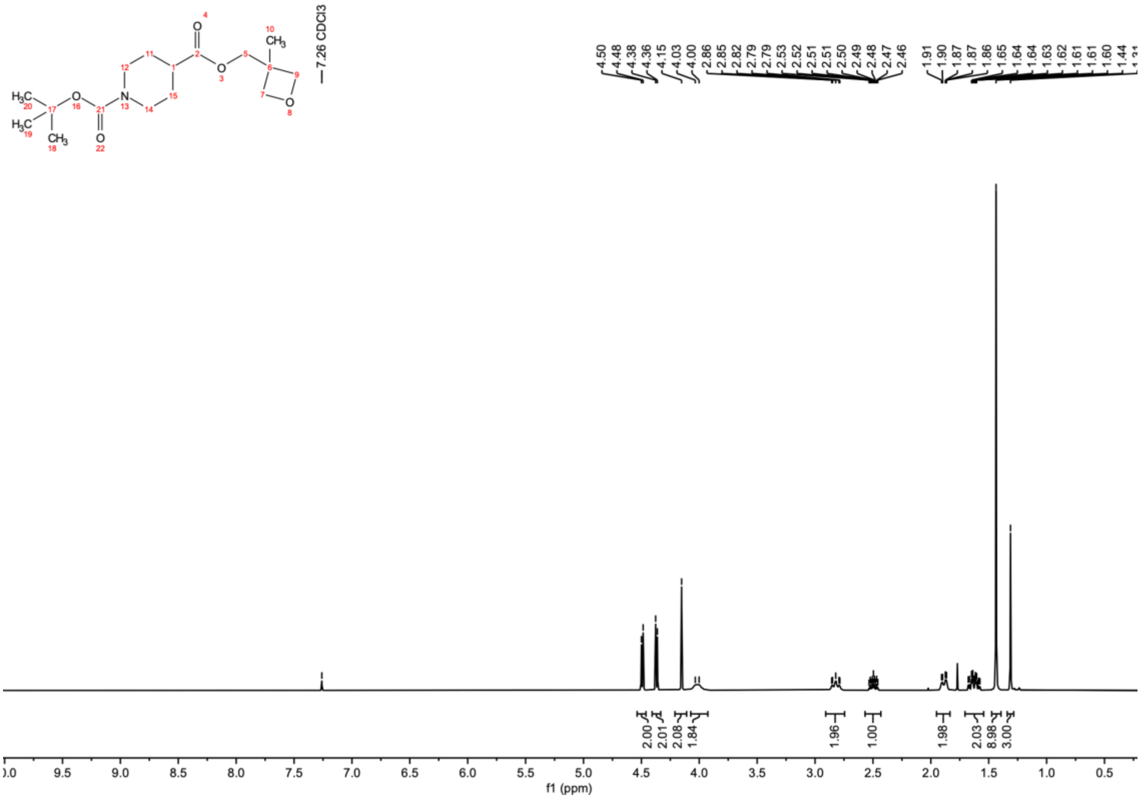

**Figure S54.** $^{1}$H-NMR of oxetane ester **2**.

DOI: 10.1039/x0xx00000x

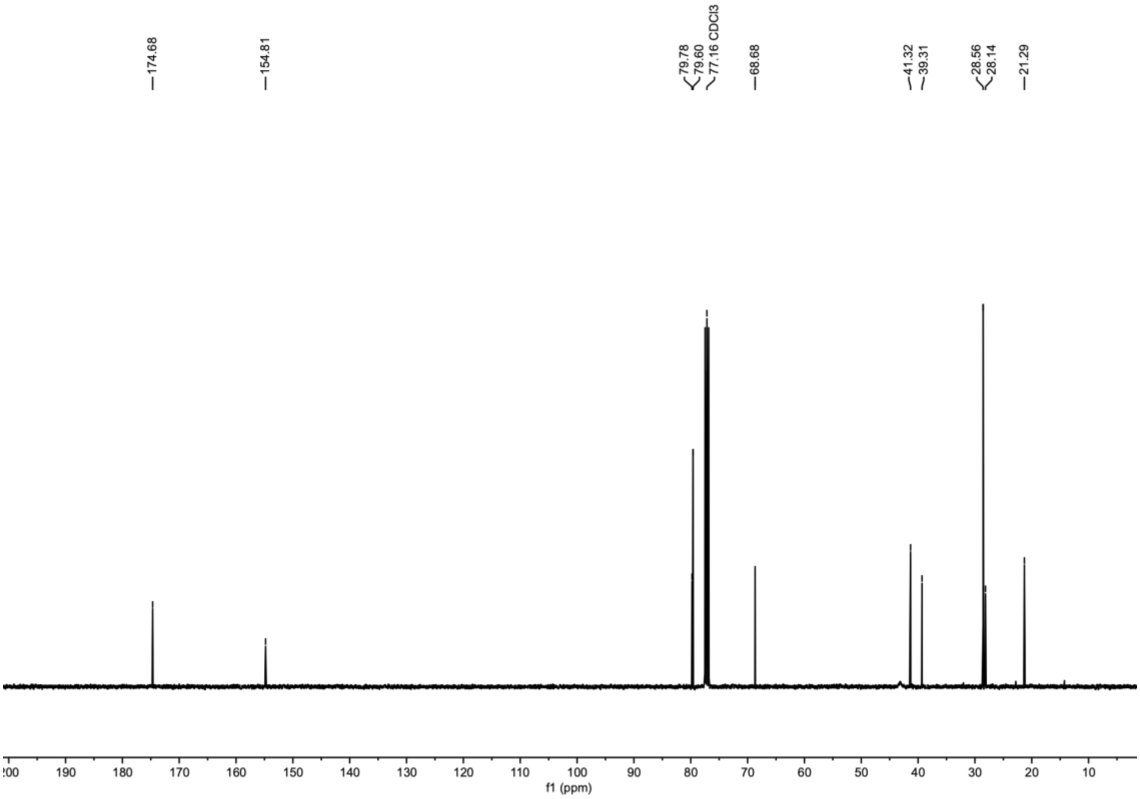

**Figure S55.** $^{13}C$-NMR of oxetane ester **2**.

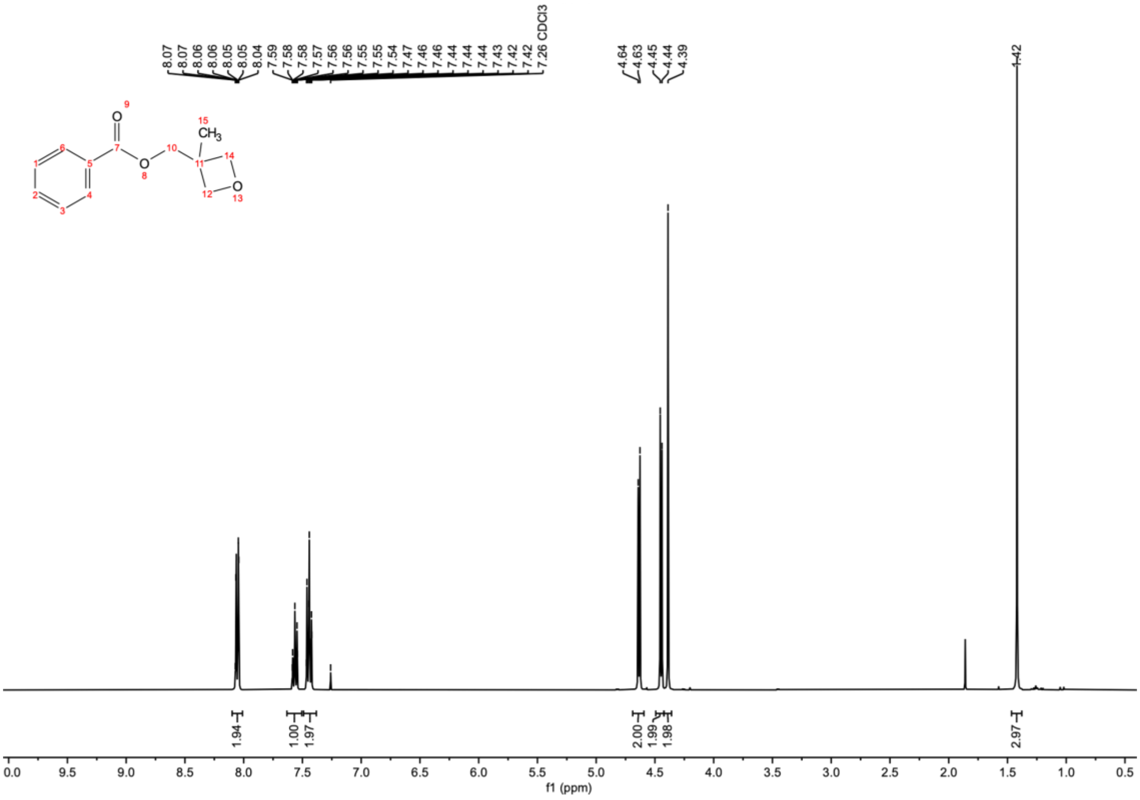

**Figure S56.** $^{1}H$-NMR of oxetane ester **3**.

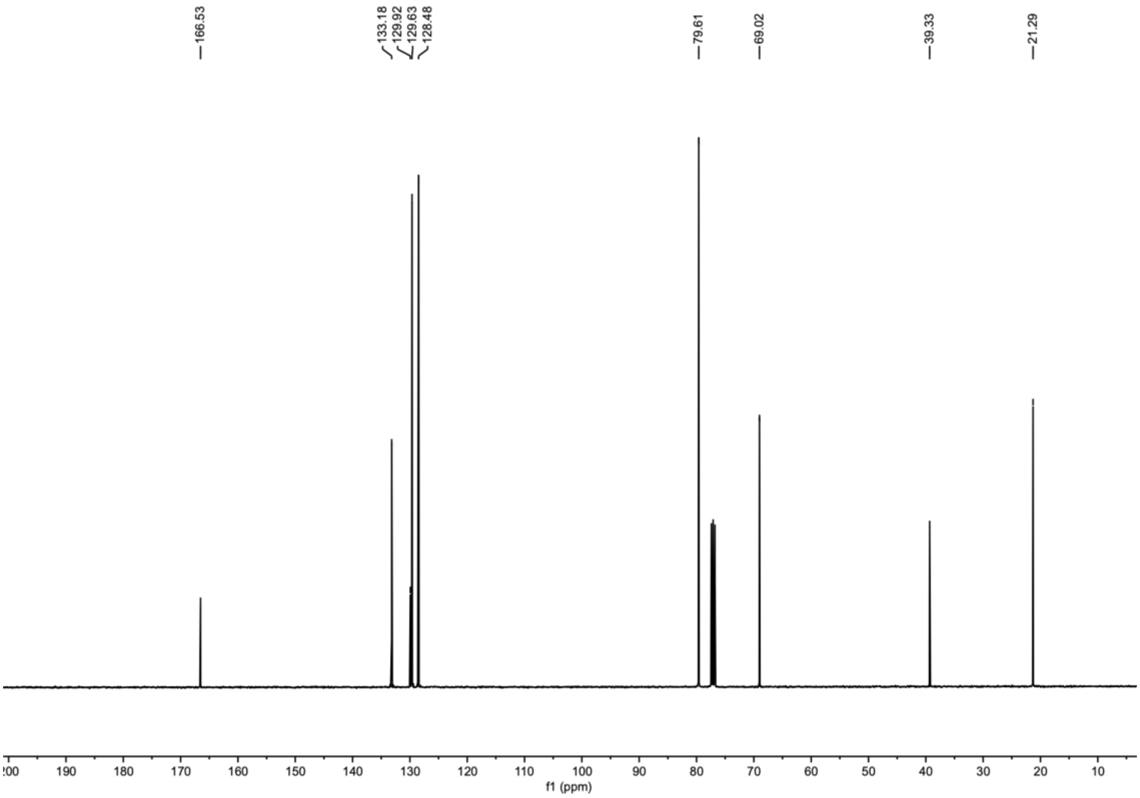


**Figure S57.** $^{13}C$-NMR of oxetane ester **3**.

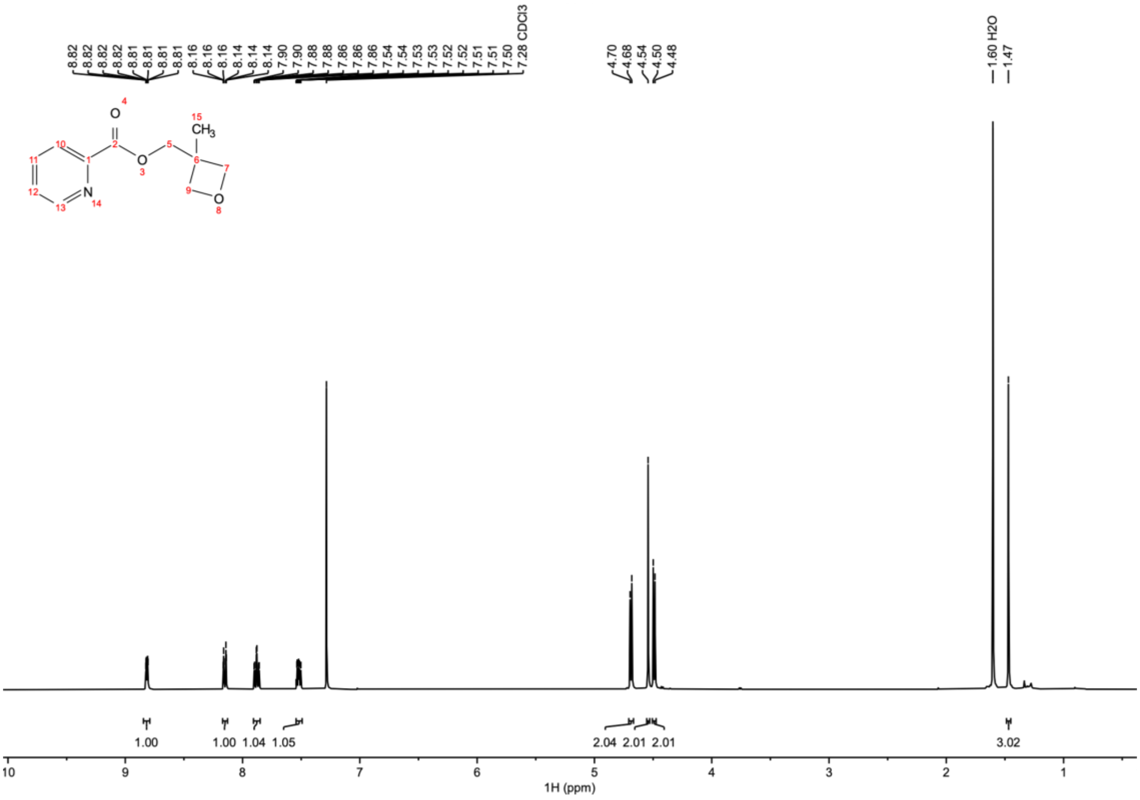


**Figure S58.** $^{1}H$-NMR of oxetane ester **4**.

DOI: 10.1039/x0xx00000x

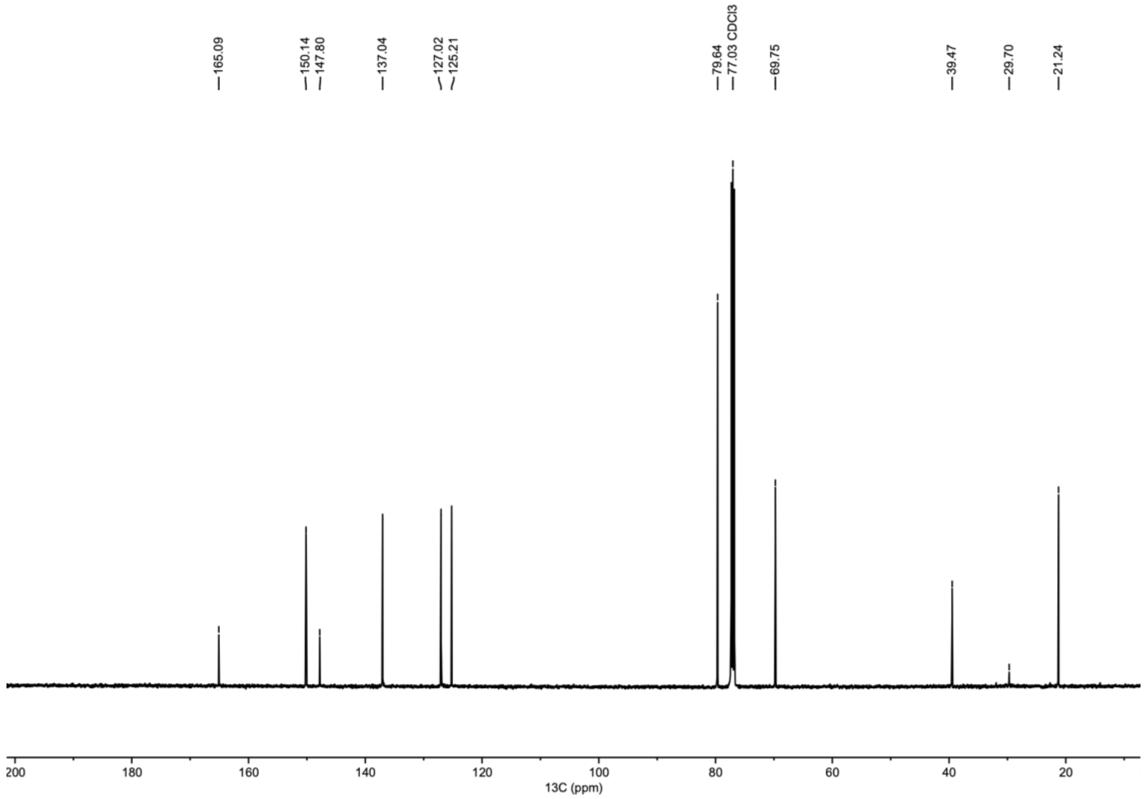


**Figure S59.** $^{13}C$-NMR of oxetane ester **4**.

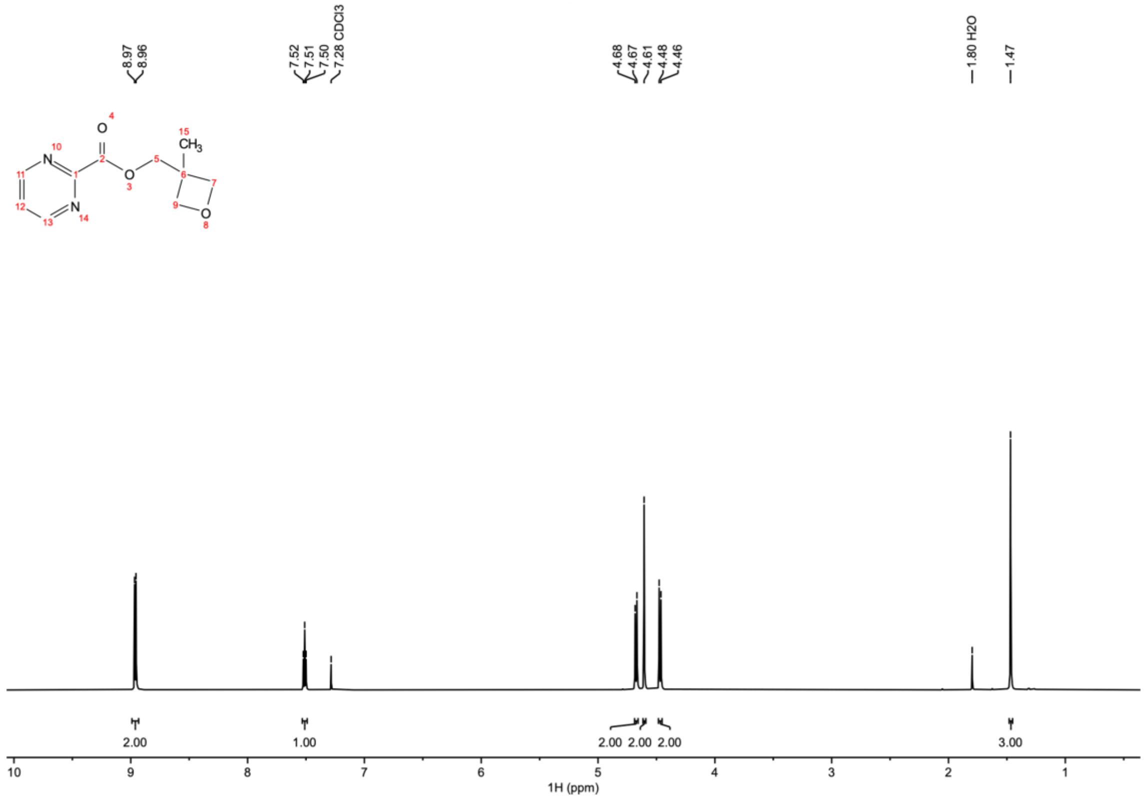


**Figure S60.** $^{1}H$-NMR of oxetane ester **5**.

 DOI: 10.1039/x0xx00000x

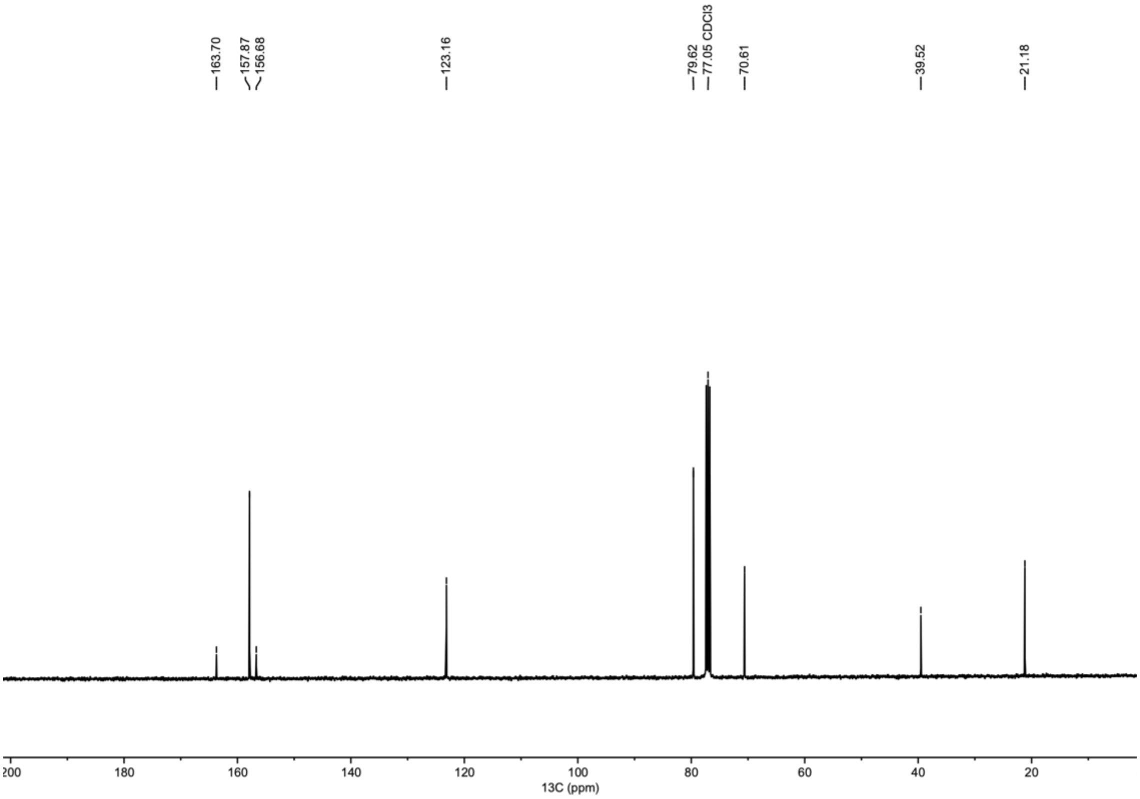


**Figure S61.** $^{13}C$-NMR of oxetane ester **5**.

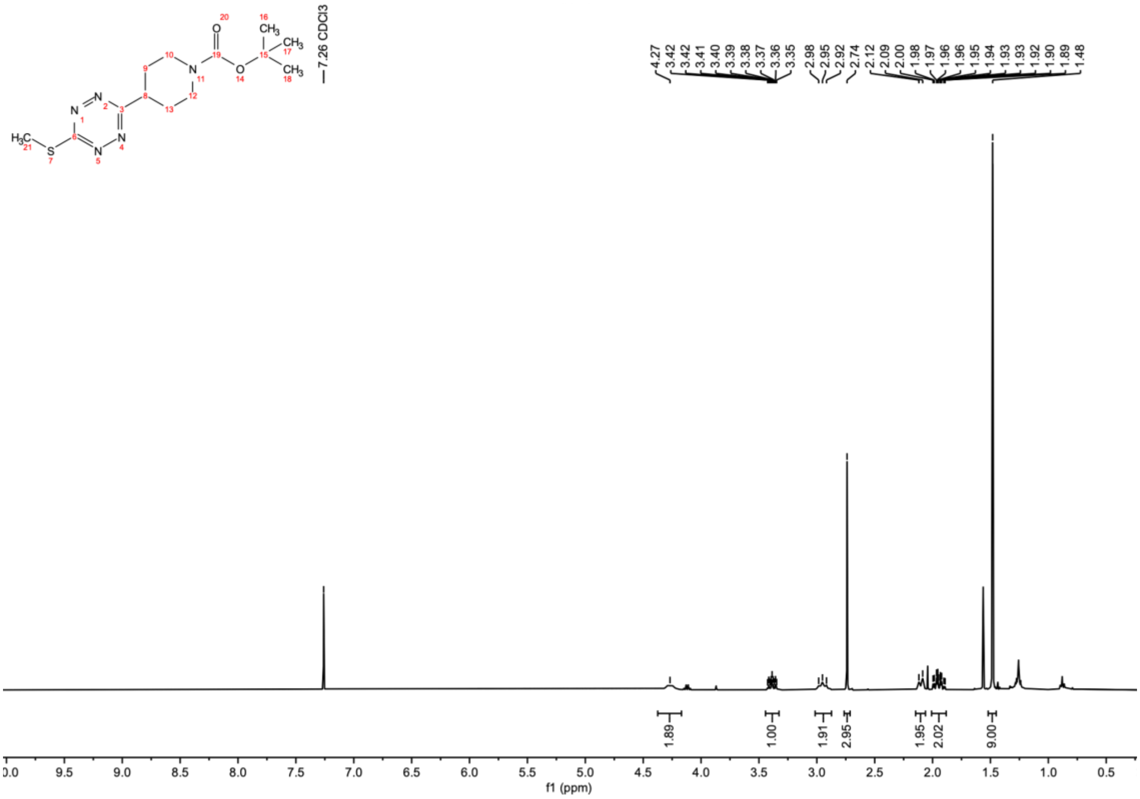


**Figure S62.** $^{1}H$-NMR of tetrazine **1Tz**.

DOI: 10.1039/x0xx00000x

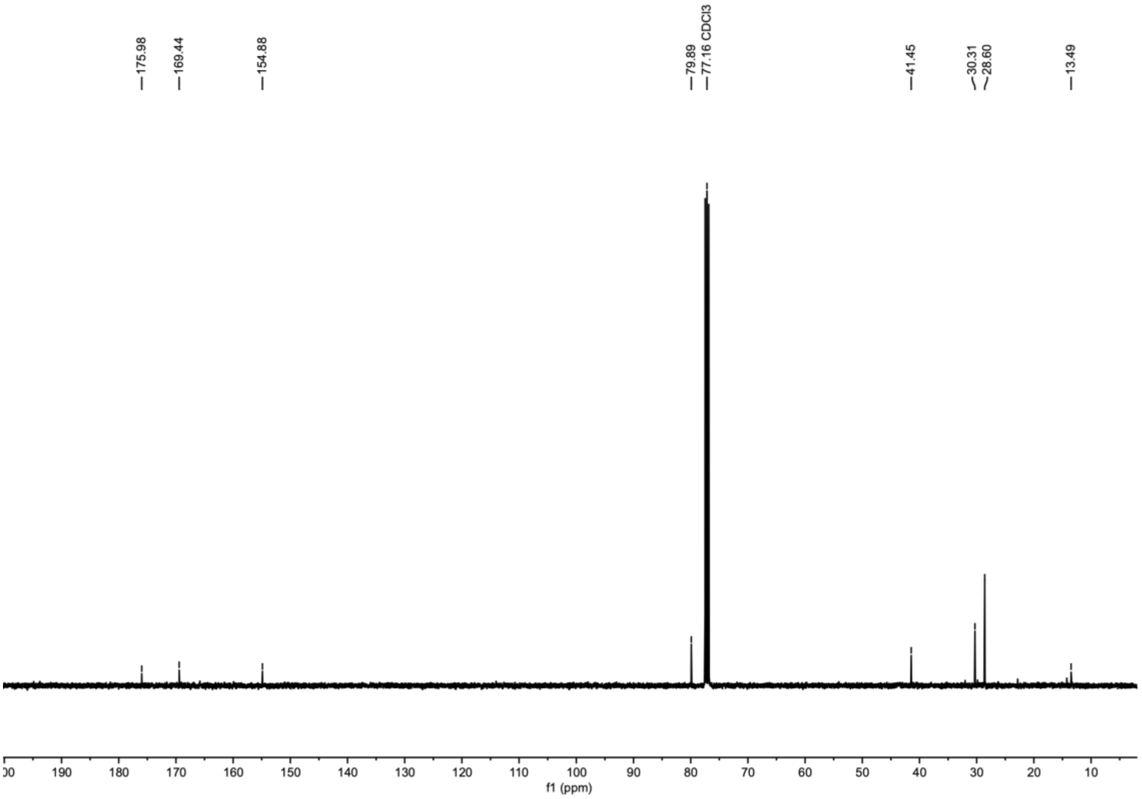


**Figure S63.** $^{13}$C-NMR of tetrazine **1Tz**.

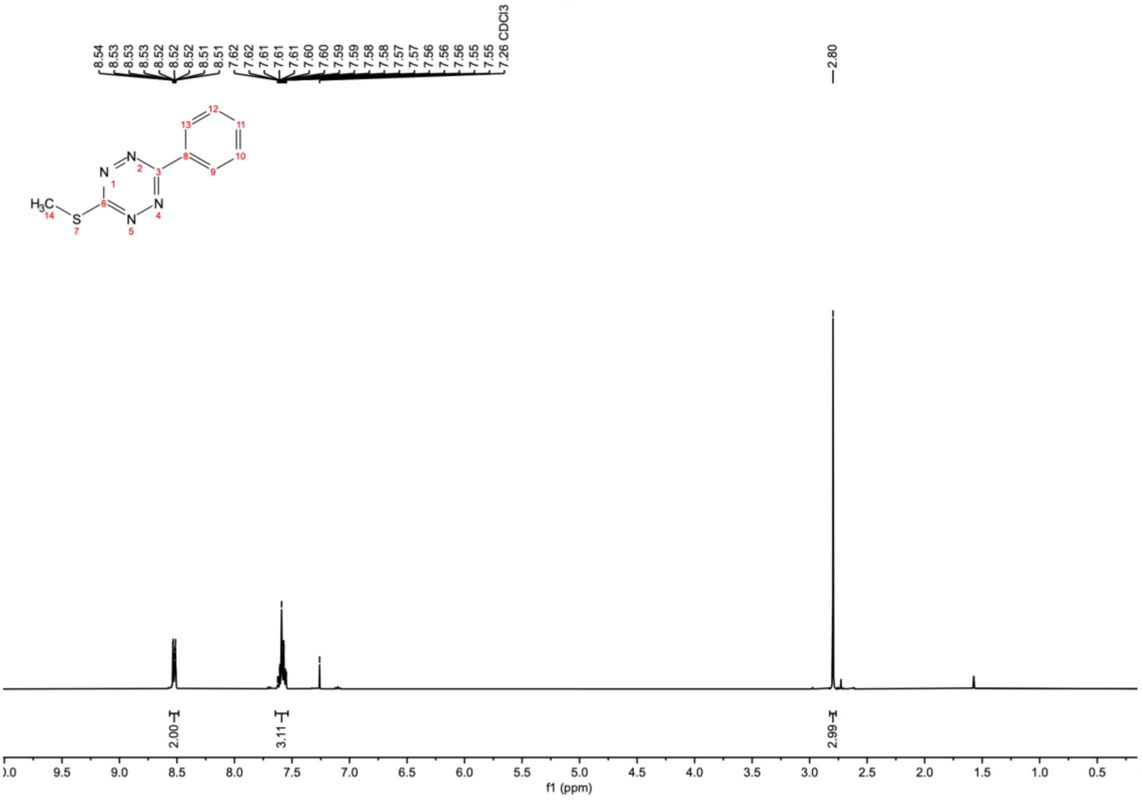


**Figure S64.** $^{1}$H-NMR of tetrazine **2Tz**.

DOI: 10.1039/x0xx00000x

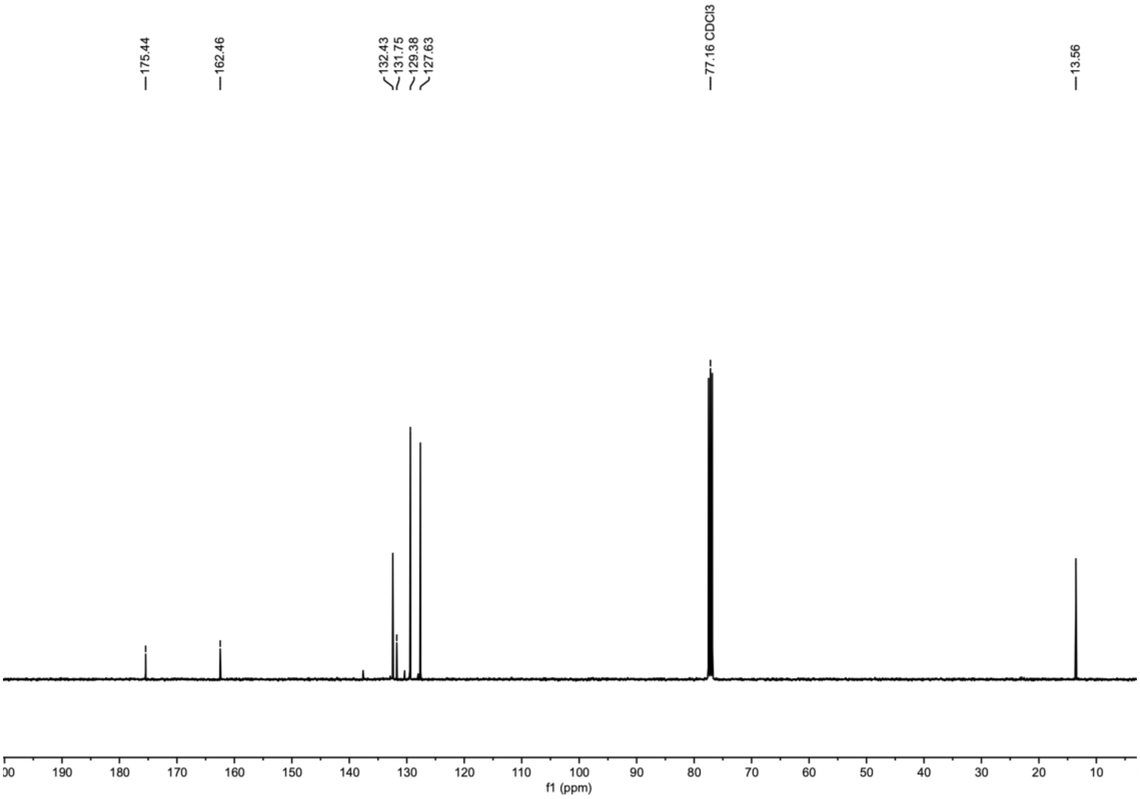


**Figure S65.** $^{13}C$-NMR of tetrazine **2Tz**.

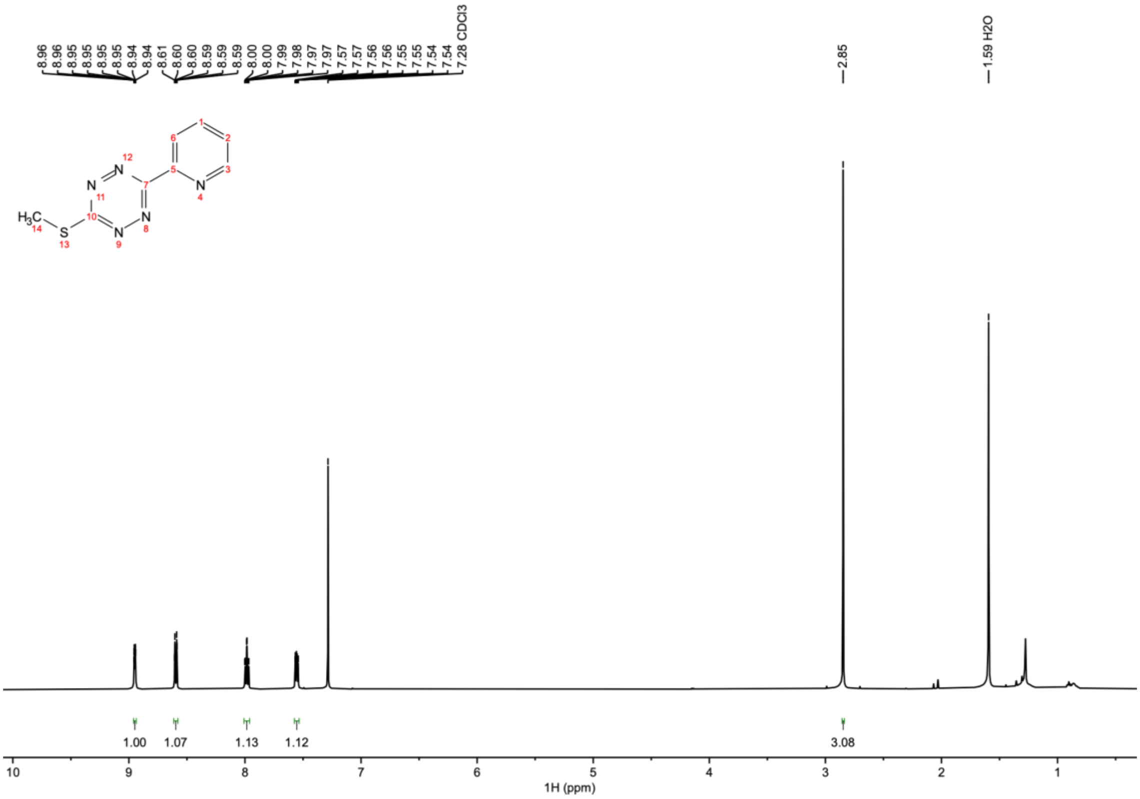


**Figure S66.** $^{1}H$-NMR of tetrazine **3Tz**.

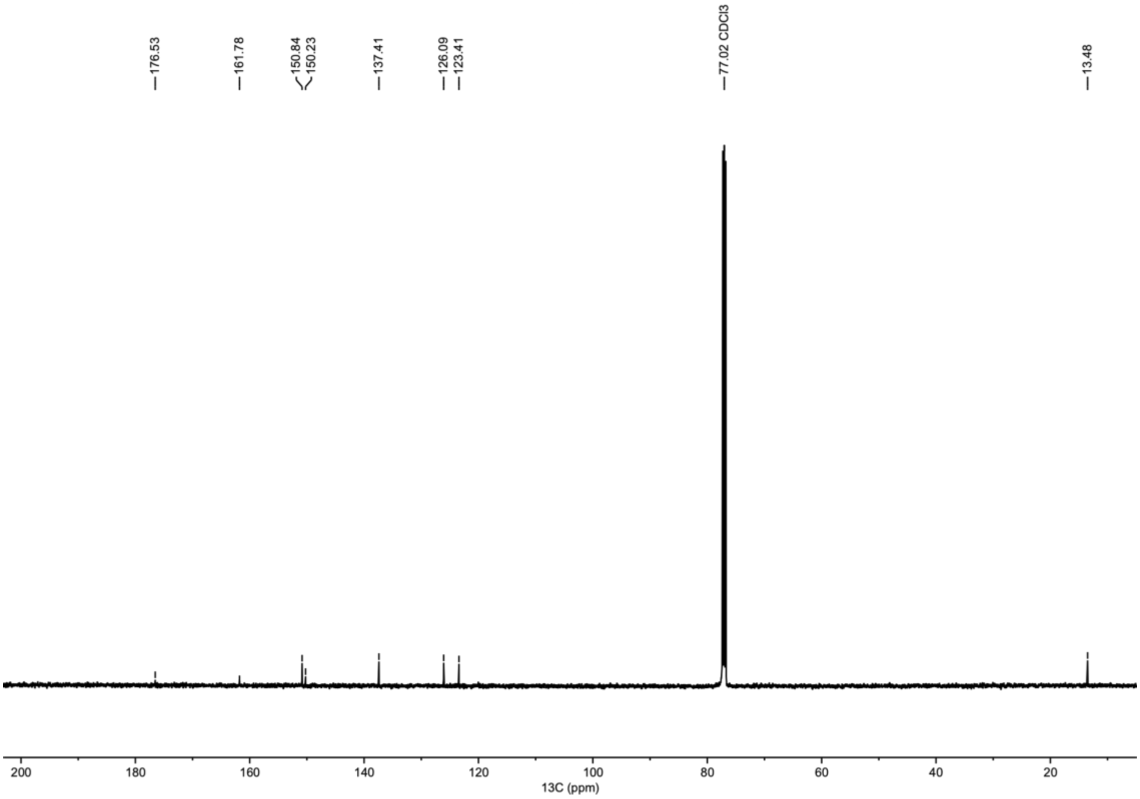


**Figure S67.** $^{13}$C-NMR of tetrazine **3Tz**.

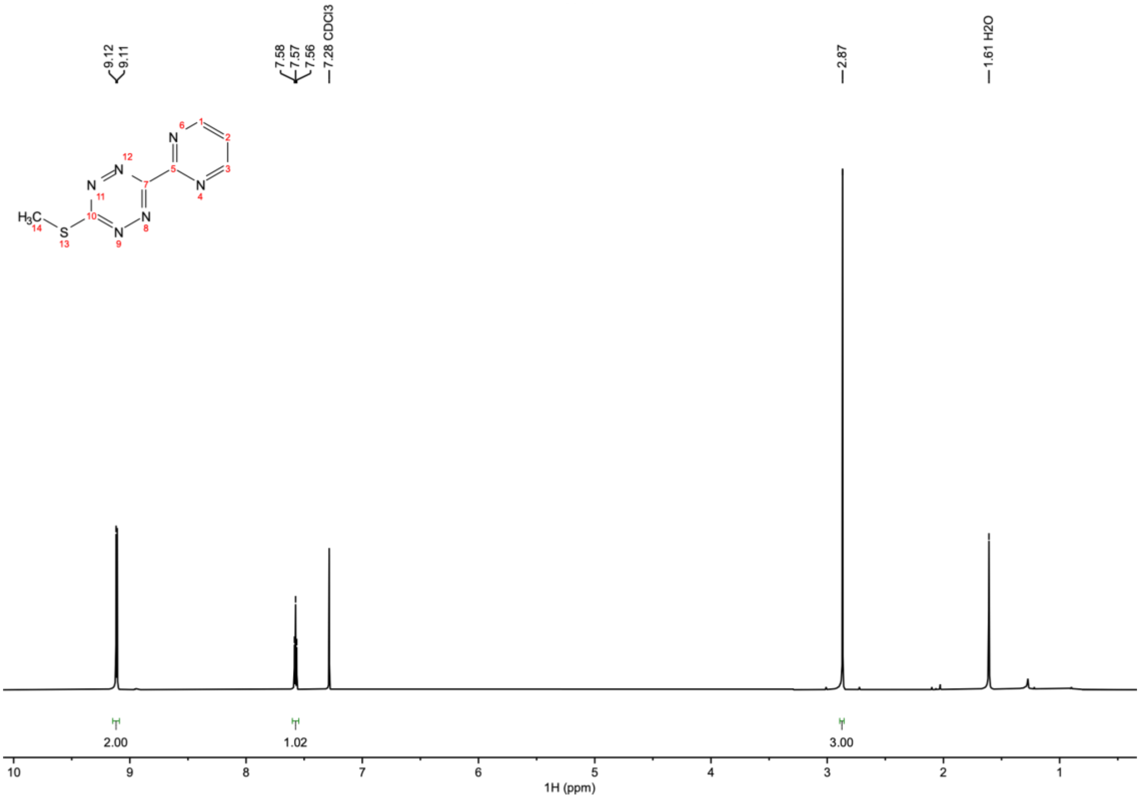


**Figure S68.** $^{1}$H-NMR of tetrazine **4Tz**.

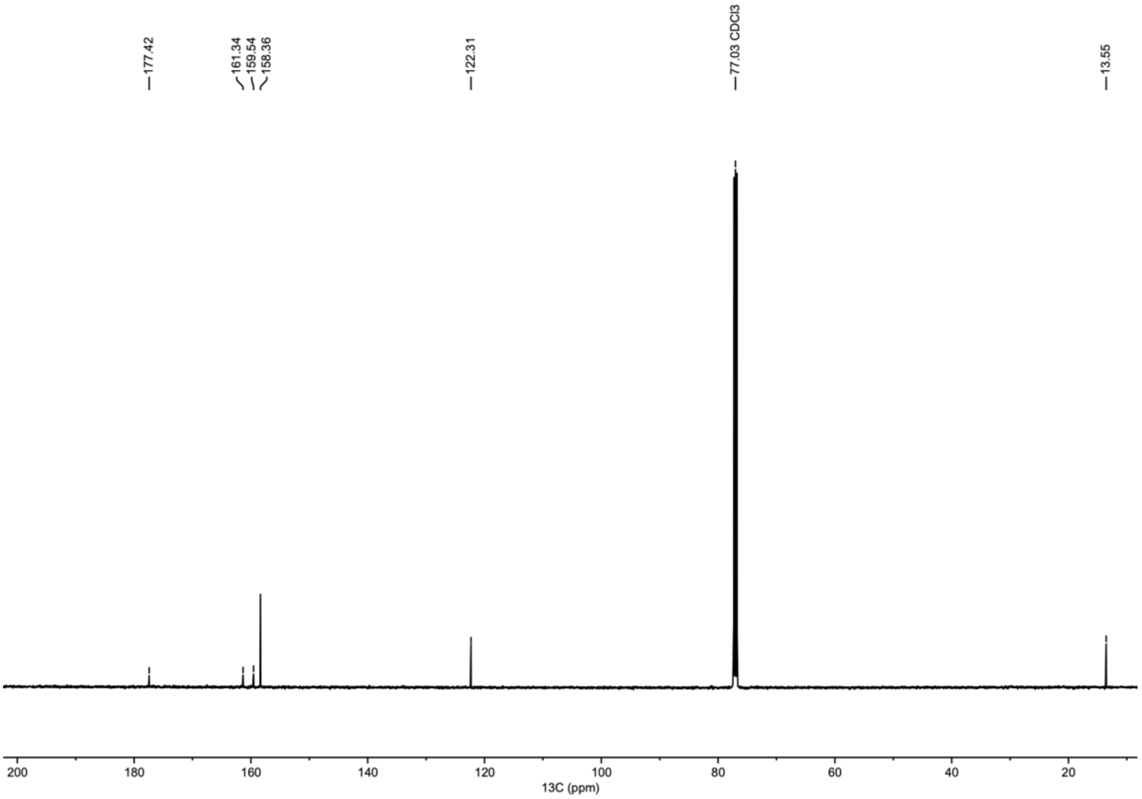


**Figure S69.** $^{13}$C-NMR of tetrazine **4Tz**.

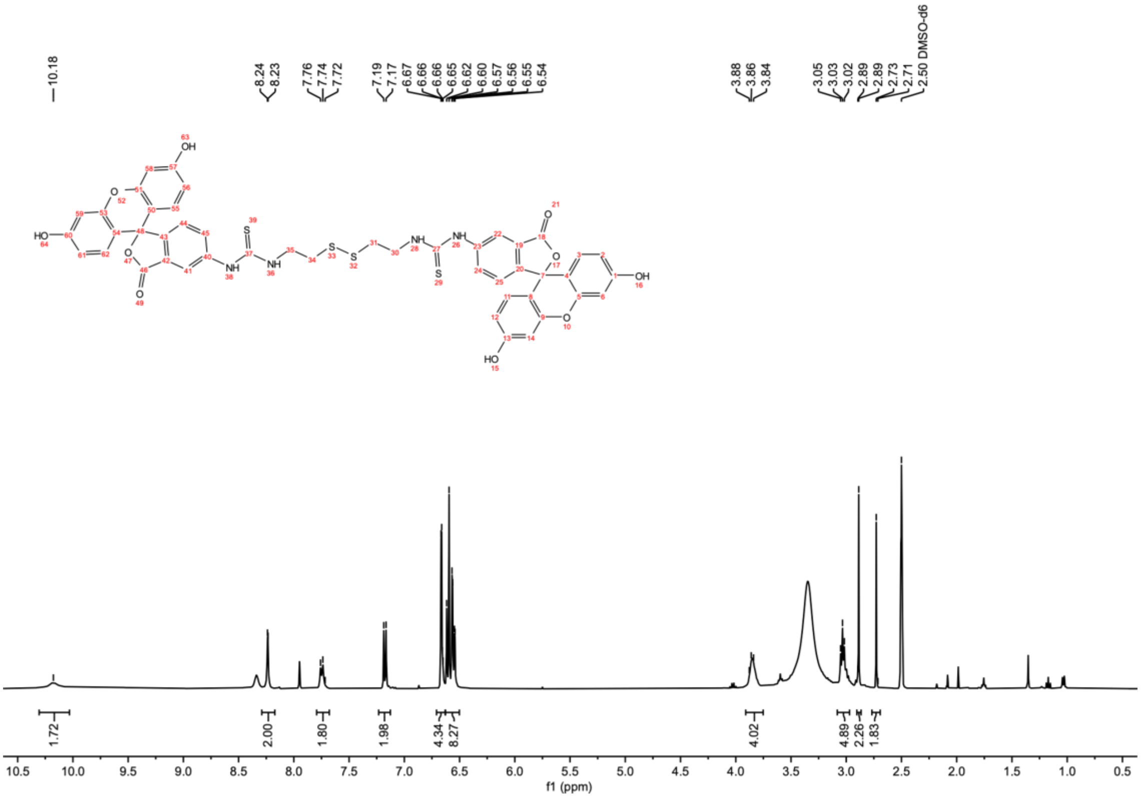


**Figure S70.** $^{1}$H-NMR of compound **6**.

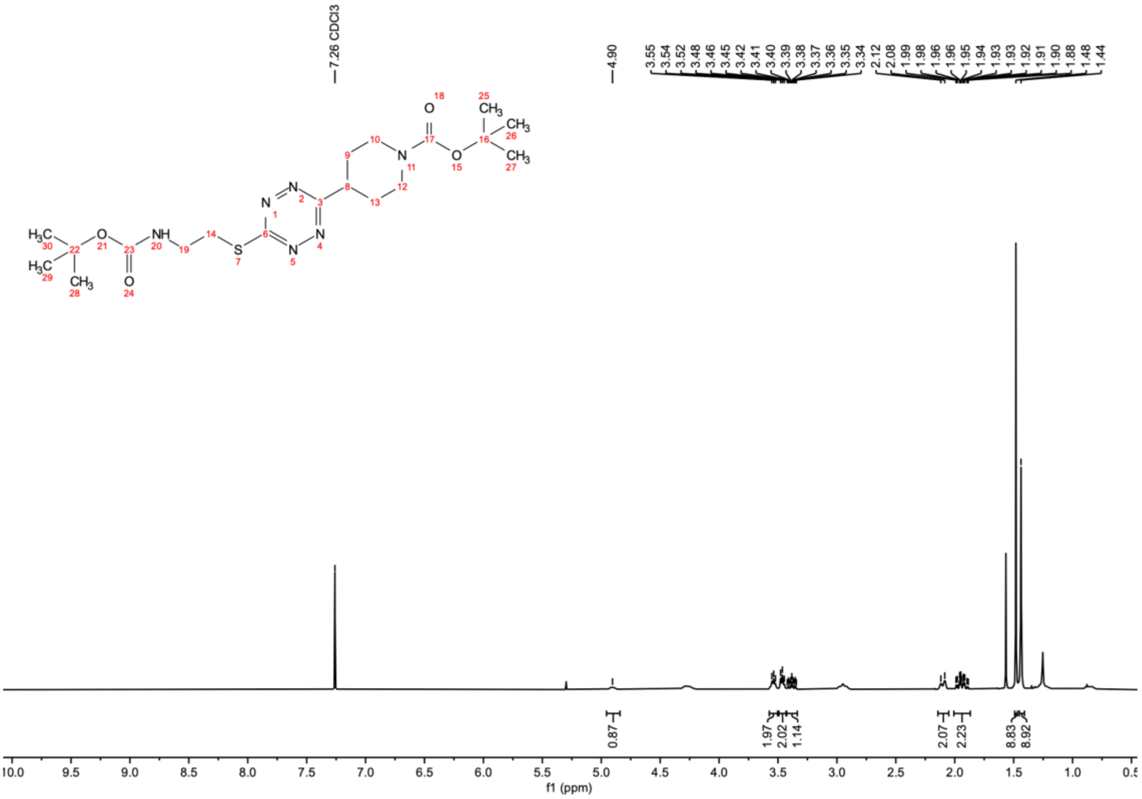

**Figure S71** $^{1}H$-NMR of tetrazine **5Tz**.

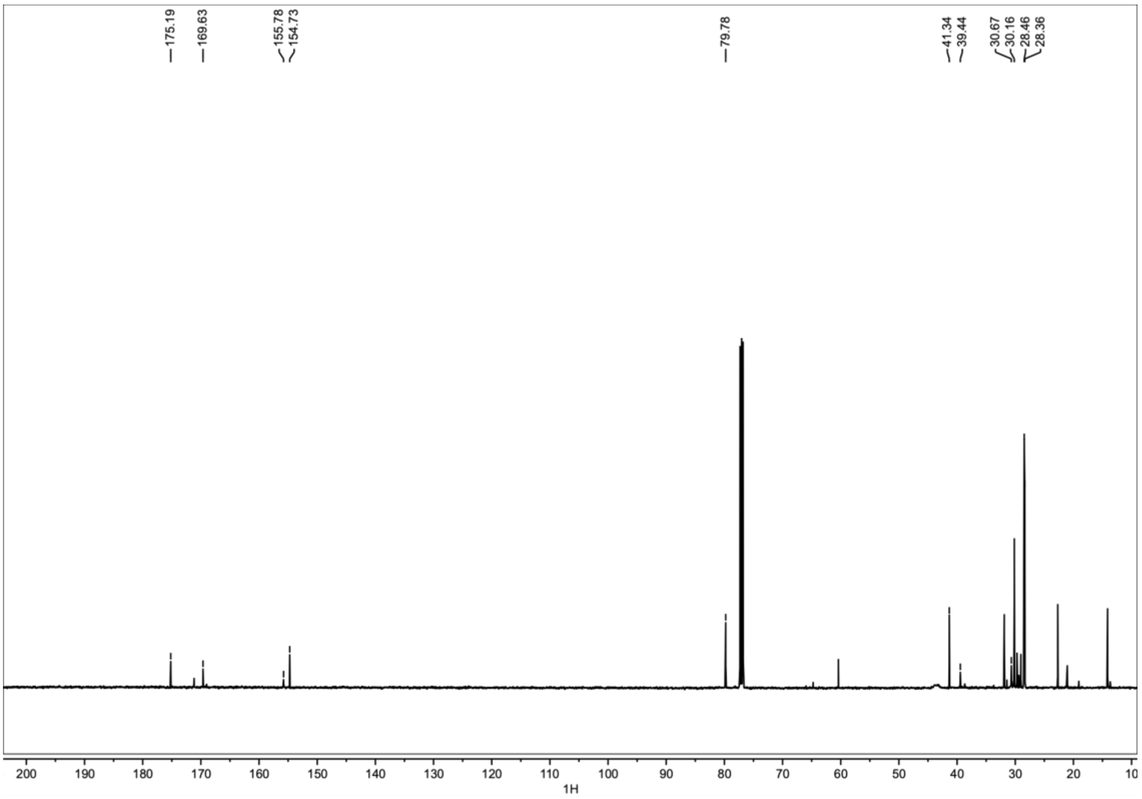

**Figure S72.** Crude $^{13}C$-NMR of tetrazine **5Tz**.

DOI: 10.1039/x0xx00000x

# References


1 Y. Xie, Y. Fang, Z. Huang, A. M. Tallon, C. W. am Ende and J. M. Fox, *Angewandte Chemie International Edition*, 2020, **59**, 16967–16973.
2 F. Saito, H. Noda and J. W. Bode, *ACS Chem. Biol.*, 2015, **10**, 1026–1033.
3 B. Bernardim, P. M. S. D. Cal, M. J. Matos, B. L. Oliveira, N. Martínez-Sáez, I. S. Albuquerque, E. Perkins, F. Corzana, A. C. B. Burtoloso, G. Jiménez-Osés and G. J. L. Bernardes, *Nat Commun*, 2016, **7**, 13128.
4 A. L. Baumann, S. Schwagerus, K. Broi, K. Kemnitz-Hassanin, C. E. Stieger, N. Trieloff, P. Schmieder and C. P. R. Hackenberger, *J. Am. Chem. Soc.*, 2020, **142**, 9544–9552.
5 M.-A. Kasper, M. Glanz, A. Stengl, M. Penkert, S. Klenk, T. Sauer, D. Schumacher, J. Helma, E. Krause, M. C. Cardoso, H. Leonhardt and C. P. R. Hackenberger, *Angewandte Chemie International Edition*, 2019, **58**, 11625–11630.
6 H. Seki, S. J. Walsh, J. D. Bargh, J. S. Parker, J. Carroll and D. R. Spring, *Chemical Science*, 2021, **12**, 9060–9068.
7 L. Xu, M. J. S. A. Silva, P. M. P. Gois, S. Ling Kuan and T. Weil, *Chemical Science*, 2021, **12**, 13321–13330.
8 H. Faustino, M. J. S. A. Silva, L. F. Veiros, G. J. L. Bernardes and P. M. P. Gois, *Chem. Sci.*, 2016, **7**, 5052–5058.
